# Biomarkers (mRNAs and Non-Coding RNAs) for the Diagnosis and Prognosis of Colorectal Cancer – From the Body Fluid to Tissue Level

**DOI:** 10.3389/fonc.2021.632834

**Published:** 2021-04-29

**Authors:** Jinhua He, Feifeng Wu, Zeping Han, Min Hu, Weida Lin, Yuguang Li, Mingrong Cao

**Affiliations:** ^1^ Department of Laboratory Medicine, Central Hospital of Panyu District, Guangzhou, China; ^2^ Department of Hepatobiliary Surgery, The First Affiliated Hospital of Jinan University, Guangzhou, China

**Keywords:** colorectal cancer, biomarkers, mRNA, ncRNA, diagnosis, prognosis

## Abstract

In recent years, the diagnosis and treatment of colorectal cancer (CRC) have been continuously improved, but the mortality rate continues to be high, especially in advanced patients. CRC patients usually have no obvious symptoms in the early stage and are already in the advanced stage when they are diagnosed. The 5-year survival rate is only 10%. The blood markers currently used to screen for CRC, such as carcinoembryonic antigen and carbohydrate antigen 19-9, have low sensitivity and specificity, whereas other methods are invasive or too expensive. As a result, recent research has shifted to the development of minimally invasive or noninvasive biomarkers in the form of body fluid biopsies. Non-coding RNA molecules are composed of microRNAs, long non-coding RNAs, small nucleolar RNAs, and circular RNAs, which have important roles in the occurrence and development of diseases and can be utilized for the early diagnosis and prognosis of tumors. In this review, we focus on the latest findings of mRNA-ncRNA as biomarkers for the diagnosis and prognosis of CRC, from fluid to tissue level.

## Introduction

Colorectal cancer (CRC) has the third highest incidence of all types of cancer worldwide, but the second highest mortality rate, with more than 1 million cases diagnosed and half a million deaths each year (1). The prognosis of CRC is related to the stage at the time of diagnosis. A quarter of patients present with lymph node-negative disease (American Joint Committee on Cancer stages I and II), and more than 50% of stage III patients have local recurrence and/or metastasis. The 5-year survival rate of patients with early CRC is 90%, whereas the 5-year survival rate of patients with distant metastasis is less than 10% (2, 3). The lack of early detection can affect the survival of CRC patients.

Reliable biomarkers that can detect CRC at an early stage could improve the prognosis, treatment response prediction, and risk of recurrence. These markers may identify susceptibility or early stages of the disease, and could also accurately identify patients at risk of disease recurrence and spread, as well as those patients who have failed systemic therapy. These patients may benefit from early active treatment, replacement therapy, and/or frequent monitoring and early detection of disease recurrence (4, 5).

### Molecular Pathogenesis of CRC

CRC develops through the gradual accumulation of genetic and epigenetic changes, leading to the transformation of normal colonic mucosa into invasive cancer. Most CRC develops from adenoma (adenoma-carcinoma sequence), and tumor transformation is deemed to take more than 10 years. Hyperplastic adenoma is the most common precancerous lesion in CRC (6). It is estimated that 20%–25% of cases have an associated genetic component, which is called familial CRC (7). Sporadic CRC is the result of a complex multi-factor process, which can lead to changes in the cell cycle of normal colonic epithelial cells.

At present, three principal molecular mechanisms are considered to lead to the onset of CRC: microsatellite instability, chromosomal instability, and CpG island methylation (8, 9). These pathways lead to the pathological transition and development of malignant tumors, accompanied by oncogenes suppressing the expression of tumor suppressor genes. This heterogeneity in the molecular pathogenesis of CRC is very important to clinical practice given that the identification of these subtypes with different subtype-specific gene markers can guide the “personalized” treatment of CRC patients (10).

## Current Diagnosis, Prognosis, and Prediction Methods of CRC

CRC is diagnosed by colonoscopy and radiography before surgery and is confirmed by biopsy or histopathological examination of surgically removed specimens (11). However, these diagnostic methods are highly invasive and costly, and cumbersome bowel preparations usually result in pain, discomfort, and financial pressure for patients. In addition, the success of colonoscopy depends on the skills and experience of the operator. Accordingly, the widespread application of colonoscopy for large-scale CRC screening has been hindered (12).

Other less invasive tests, such as the stool occult blood test and serum tests for tumor markers such as carcinoembryonic antigen (CEA) and carbohydrate antigen 19-9 (CA19-9) are commonly used in clinical practice, but their sensitivity and specificity are poor and their value is limited (13). Therefore, innovative large-scale screening programs have been established using feces or body fluids targeting mRNA expression, gene mutations (such as KRAS, adenomatous polyposis coli [APC], and p53), microsatellite instability, or methylated promoter regions (14). At present, a large amount of research has been carried out worldwide to identify molecular markers based on DNA, RNA, or proteins to develop novel, non-invasive blood and stool CRC biomarker detection methods (15–20).

The APC gene is mutated in CRC, and its inactivation is considered to be a key early genetic change in CRC (21). DNA sequencing and RT-PCR analysis of APC gene expression and APC gene mutations in tumor tissues of 195 CRC patients found that 66 (33.8%) of 195 tumor tissues contained APC gene mutations, and indicated that APC gene mutations can be used as a marker for the clinical prognosis of CRC.

p53 (encoded by TP53) is involved in DNA damage repair, cell cycle regulation, apoptosis, and cellular senescence (22). The role of p53 inactivation in the progression and prognosis of CRC has been studied extensively, but still remains unclear. Loss of this gene is associated with a poor prognosis in CRC patients (23).

Epidermal growth factor receptor (EGFR) is a protein found on cells that plays a vital role in promoting cell growth. In gastric, breast, endometrial cancer and CRC,.EGFR overexpression is associated with reduced recurrence-free or overall survival rates (24). Cetuximab inhibits EGFR-mediated signaling by blocking its binding to endogenous ligands, and patient resistance to cetuximab is also associated with the EGFR pathway. In conclusion, EGFR is a key prognostic factor for CRC patients (25).

At present, for anti-EGFR antibody-based treatments, as well as the response to cetuximab and panitumumab, mutations in the KRAS gene are the most commonly used marker (26). The KRAS proto-oncogene encodes a small G protein (guanosine triphosphate-/guanosine diphosphate-binding protein) in the PI3K/PTEN/AKT and RAF/MEK/ERK signaling pathways downstream of EGFR. Most KRAS-activating mutations (~90%) are found in codons 12 and 13 of exon 1, and nearly 5% of mutations occur in codon 16 of exon 2 (27, 28). It has been suggested that KRAS gene mutation analysis combined with phosphatidylinositol-4,5-bisphosphate 3-kinase catalytic subunit alpha mutation analysis can be used as a prognostic marker for CRC before anti-EGFR treatment is given (8). At present, the diagnostic test markers for CRC also include circulating tumor cells, CEA and CA19-9, cell-free nucleic acid, serum DNA, and other circulating DNA methylation biomarkers (vimentin, nerve growth factor receptor, septin 9 and transmembrane protein with EGF-like and two follistatin-like domains 2, p16, APC, mutL homolog 1, helicase-like transcription factor, and death-associated protein kinase 1), mRNA, and non-coding RNA (ncRNA) (8).

## Candidate RNA Molecules as Biomarkers for CRC

### mRNAs as Biomarkers for CRC

mRNAs are single-stranded ribonucleic acid molecules that are transcribed from a strand of DNA as a template, carry genetic information, and can guide protein synthesis (29, 30). The levels of free baculoviral IAP repeat-containing 5 (BIRC5) mRNA are significantly increased in the serum of CRC patients, with a sensitivity of 84.8% and a specificity of 80.0%. In addition, when BIRC5 mRNA is combined with CEA, its diagnostic performance is significantly improved. Patients with high levels of BIRC5 mRNA have a worse prognosis than those with low levels. BIRC5 mRNA is a non-invasive molecular biomarker used in the diagnosis of CRC and has a higher diagnostic efficacy compared with CEA (31). B-lymphoma Moloney murine leukemia virus insertion region-1 (BMI1), a member of the Polycomb group family of proteins, is involved in axial patterning, hematopoiesis, regulation of proliferation, and senescence, BMI1 can be utilized as a non-invasive biomarker for monitoring occult metastasis and predicting the occurrence of distant metastases in CRC (32).

Hereditary non-polyposis CRC (HNPCC) is caused by functional defects of mismatch repair genes, including MLH1 and mut S homolog 2. MLH1 mRNA levels in peripheral blood have a high diagnostic value for HNPCC (33). The sensitivity of MLH1 mRNA levels to distinguish HNPCC from a control group is 81.3%, with a specificity of 86.7%.

The expression of cytokeratin 19, cytokeratin 20, and coronary cell cyclase C mRNAs in peripheral blood can be used for the diagnosis of non-metastatic CRC; when used in combination, their expression has a sensitivity and specificity of 88% and 68%, respectively. Coronary cell cyclase C, with a specificity of 100%, is considered a specific marker for the detection of CRC (34). The detection of cytokeratin 20 mRNA expression in the serum of CRC patients has extremely high specificity as a marker for the diagnosis of CRC (35). The expression of organic anion transporting polypeptide 1B3 (Ct-OATP1B3) mRNA in CRC tissue and adjacent tissue is related to the overall survival rate of CRC patients. At the same time, Ct-OATP1B3 mRNA is present in extracellular vesicles derived from CRC patients and can be detected in serum samples. The detection of Ct-OATP1B3 mRNA in CRC-derived extracellular vesicles as a diagnostic biomarker is worthy of further study (36).

The expression of related genes detected in platelets of CRC patients can also be used in the diagnosis of CRC. An increase in expression of TIMP metallopeptidase inhibitor 1 mRNA in platelets of CRC patients has a much higher receiver operating characteristic curve (0.958; 95% confidence interval [CI], 0.936–0.980) than CEA (0.765; 95% CI, N/A) and CA19-9 (0.612; 95% CI, N/A), indicating that the expression of TIMP metallopeptidase inhibitor 1 mRNA in platelets could be used as a non-invasive biomarker (37).

The newly discovered prognostic biomarker solute carrier family 35 member D3 is highly expressed in the cancerous tissue of CRC patients. It is associated with CEA cell adhesion molecule 5, kallikrein-related peptidase 6, and mucin 2, and a combined construction formula can be used to identify people at risk. CEA cell adhesion molecule 5 is suggested to be the common denominator of three biomarkers (kallikrein-related peptidase 6, solute carrier family 35 member D3, and mucin 2). This formula produces 5 categories (-1, 0, 1, 2, and 3). Categories -1 and 0 suggest a good prognosis, categories 1 and 2 suggest a relatively poor prognosis, and category 3 suggests a poor prognosis. This approach, which converts data into a simple formulation based on the ratio of several biomarkers, could provide useful tools for the postoperative treatment of CRC patients and for the future development of new therapies (38).

### MicroRNAs (miRNAs) as Biomarkers for CRC

Approximately 70%–90% of the human genome is transcribed into RNA, but most RNA transcripts are non-coding, and only 2% of the genome encodes proteins (39). ncRNAs are important molecules that regulate the expression of genes at different stages such as the epigenetic, transcription, and post-transcription levels (40, 41). ncRNAs are divided into three main categories: short/small ncRNAs, long ncRNAs (lncRNAs), and circular RNAs (circRNAs). Short/small ncRNAs are also divided into three main sub-categories: miRNAs, short interfering RNAs, and PIWI-interacting RNAs (piRNAs). Other types of ncRNAs are found universally in all cell types, which may be considered as housekeeping RNAs, including transfer RNAs (tRNAs) and small nucleolar RNAs (snoRNAs) (1).

miRNAs are a class of endogenous ncRNAs of 20–25 nucleotides in length that are found in eukaryotes and have regulatory functions. Mature miRNAs are produced from long primary transcripts by a series of nucleases and then assembled into an RNA-induced silencing complex, which recognizes target mRNAs through base complementary pairing and guides the silencing complex to degrade target mRNAs or inhibit the translation of target mRNAs according to different degrees of complementation (42, 43). In the past 5 years, extensive research has been conducted on miRNAs as clinically relevant biomarkers for CRC (**Table 1**).

**Table 1:** MicroRNAs as potential biomarkers for colorectal cancer.

MicroRNA	Expression	Sample	Biomarker	Reference
miR-21	↑	Tissue/Plasma exosomes	Diagnostic/Prognostic	(1–4)
miR-23a	↑	Serum/Plasma exosomes	Diagnostic	(3, 5)
miR-17-5p	↑	Plasma/Tissue/Exosomes	Diagnostic/Prognostic	(6, 7)
miR-150-5p	↓	Serum	Diagnostic	(8, 9)
miR-92a	↑	Plasma exosomes	Diagnostic	(3, 4)
miR-29a	↑	Serum/Exosomes/Stool	Diagnostic	(4, 10)
miR-122	↑	Serum	Diagnostic/Prognostic	(11)
miR-199a/b-3p	↑	Serum	Prognostic	(12)
miR-199a-5p	↑	Serum	Prognostic	(12)
miR-199b-5p	↑	Serum	Prognostic	(12)
miR-99b-5p	↓	Serum	Diagnostic	(8)
miR-4461	↓	Serum	Diagnostic/Therapeutic	(13)
miR-92b	↓	Serum	Diagnostic	(14)
miR‐320d	↑	Serum	Diagnostic	(15)
miR-301a	↑	Serum	Diagnostic	(5)
miR-181a-5p	↑	Plasma/Tissue/Exosomes	Diagnostic	(6)
miR-18a-5p	↑	Plasma/Tissue/Exosomes	Diagnostic	(6)
miR-18b-5p	↑	Plasma/Tissue/Exosomes	Diagnostic	(6)
miR-548c-5p	↓	Plasma exosomes	Diagnostic/Prognostic	(16)
miR-27a	↑	Plasma exosomes	Diagnostic/Prognostic	(17)
miR-130a	↑	Plasma exosomes	Diagnostic/Prognostic	(17)
miR-92a-3p	↑	Plasma exosomes	Prognostic	(7)
miR-6803-5p	↑	Plasma exosomes	Diagnostic/Prognostic	(18)
miR-6869-5p	↓	Plasma exosomes	Prognostic	(19)
miR-125a-3p	↑	Plasma exosomes	Diagnostic	(20)
miR-96-5p	↓	Tissue/Plasma exosomes	Diagnostic/Therapeutic	(21)
miR-149	↓	Tissue/Plasma exosomes	Diagnostic/Therapeutic	(21)
miR-19a-3p	↑	Tissue/Plasma exosomes	Diagnostic	(22)
miR-21-5p	↑	Tissue/Plasma exosomes	Diagnostic	(22)
miR-425-5p	↑	Tissue/Plasma exosomes	Diagnostic	(22)
miR-1246	↑	Plasma exosomes	Diagnostic	(3)
miR-4772-3p	↓	Plasma exosomes	Prognostic	(23)
miR-19a	↑	Serum/Exosomes	Prognostic	(24)
miR-34a-5p	↓	Tissue	Prognostic	(25)
miR-132	↓	Tissue	Prognostic	(26)
miR-199b	↓	Tissue	Prognostic	(27)
miR-145	↓	Tissue/Serum	Prognostic	(1)
miR-223	↓	Stool	Diagnostic	(10)
miR-224	↓	Stool	Diagnostic	(10)
miR-195-5p	↓	Tissue/Cell lines	Prognostic	(28)
miR-145-5p	↓	Tissue	Diagnostic/Therapeutic	(29)

References showed in **Supplementary Material 1**.

miRNAs are present in CRC tumor tissue, feces, and various body fluids (plasma, serum, exosomes, and urine). miR-143 and miR-145 expression is significantly downregulated in CRC tissue and both have a role in the pathogenesis of CRC (44). miR-143 overexpression can reduce the expression of KRAS protein and inhibit cell proliferation (45). The inhibitory effect of miR-143 on KRAS expression represses the phosphorylation of extracellular regulatory protein kinase 1/2 and then stimulates cell proliferation. miR-143 downregulation may promote tumor development (46). miR-143 can enhance the sensitivity of KRAS mutant CRC cells to paclitaxel treatment (47). miR-145 inhibits cell viability, migration, and invasion by targeting the tumor suppressor candidate gene 3 in CRC cells (48). miR-145 expression is significantly higher in CRC patients with lymph node metastasis compared with patients without lymph node metastasis, and it plays an important role in advanced CRC (49). Low miR-145 expression is linked to poor prognosis; patients with low miR-145 expression have a 1.92-fold higher short-term overall survival risk than patients with high expression (45). Given the lack of abundant miR-143 and miR-145 expression data in the global population, further large-scale, well-designed, multicenter prospective studies are needed to confirm these findings before miR-143 and miR-145 can be used as disease progression biomarkers to predict CRC survival outcomes.

miR-21 is an oncogene that is upregulated in almost all malignant tumors, including CRC tumor tissue (50), and it is steadily upregulated in CRC patient serum (51). High serum levels of miR-21 and miR-92a may be potential biomarkers for the early detection of CRC and advanced adenoma (52). Programmed cell death 4 and phosphatase and tension homolog (PTEN) levels are negatively correlated with the expression of miR-21 in CRC tissue and cells (53). In serum samples from 200 CRC patients, 50 advanced adenoma patients, and 80 healthy controls, the area under the receiver operating characteristic curve (AUC) of miR-21 was 0.802, and that of miR-92a was 0.786 (54). miR-92a plays a role in CRC by targeting the tumor suppressor PTEN, and high miR-92a expression is significantly correlated with tumor, node, metastasis (TNM) staging, lymph node metastasis, and distant metastasis (55).

Exosomes are lipid vesicles with a diameter of 40-100 nm that were first discovered in sheep reticulocytes in 1983 (56). Exosomal miR-21 levels are an independent prognostic factor of overall survival and disease-free survival in TNM stage II/III CRC patients and overall survival in TNM stage IV patients (51). The growth rate of CRC is related to the concentration of miRNAs in exosomes. miR-21, miR-92a, and miR-1246 overexpression in exosomes promotes the proliferation of cancer cells, whereas miR-23a and miR-92a overexpression inhibits apoptosis in cancer cells (57).

miRNAs are sufficiently stable to be detected in stool samples because they are protected in exosomes (58). miR-21 is not only highly expressed in the serum of patients with CRC but can also be detected at high levels in stool samples. High levels of miR-92a are found in the stool samples from CRC patients (59). Twelve upregulated miRNAs (miR-7, miR-17, miR-20a, miR-21, miR-92a, miR-96, miR-106a, miR-134, miR-183, miR-196a, miR-199a-3p, and miR-214) and eight downregulated miRNAs (miR-9, miR-29b, miR-127-5p, miR-138, miR-143, miR-146a, miR-222, and miR-938) can distinguish different TNM stages with high sensitivity and specificity (60). miR-135b and miR-31 were found to be significantly upregulated in CRC and advanced adenoma as compared with their adjacent normal tissues. the expression of miR-135b correlated positively with stages of lesions, with more advanced lesions having the highest miRNA level. The expression levels of miR-135b in feces can be used to distinguish different stages of CRC (61). In extracellular vesicles isolated from peritoneal lavage fluid, 210 miRNAs were found to be significantly dysregulated; the top 10 miRNAs with an AUC value higher than 0.95 were miR-199b-5p, miR-150-5p, miR-29c-5p, miR-218-5p, miR-99a-3p, miR-383-5p, miR-199a-3p, miR-193a-5p, miR-10b-5p, and miR-181c-5p (62).

### lncRNAs as Biomarkers for CRC

lncRNAs are special ncRNA molecules of more than 200 nucleotides in length (63). lncRNAs exert regulatory functions at different levels of gene expression, including chromatin modification, transcription, and post-transcription (64). In cancer, lncRNAs may promote cell proliferation, invasion, and development, induce angiogenesis, and promote cell resistance to apoptosis (65). lncRNAs are abnormally expressed in various types of cancer cells and play vital roles in common cancer characteristics (66). In recent years, numerous reports have indicated that dysregulation of lncRNA expression has been found in the tumor tissue, blood, and exosomes of CRC patients (67–69). These dysregulated lncRNAs can be used as new biomarkers for the diagnosis, treatment, and prognosis of CRC patients (**Table 2**).

**Table 2:** Potential long non-coding RNAs as biomarkers for the diagnosis and prognosis of colorectal cancer.

LncRNA	Expression	Sample	Biomarker	Reference
CCAT1	↑	Tissue/Plasma	Diagnostic/Prognostic	(1–4)
HOTAIR	↑	Tissue/Cell lines	Diagnostic/Therapeutic	(5–7)
NEAT1	↑	Cell lines	Diagnostic/Therapeutic	(8–10)
PVT1	↑	Tissue	Diagnostic/Prognostic	(11–13)
MALAT1	↑	Tissue/Cell lines		(3, 14)
UAC1	↑	Tissue/Cell lines/Exosomes	Diagnostic/Prognostic	(11, 15)
BCYRN1	↑	Tissue/Cell lines	Prognostic/Therapeutic	(16, 17)
CCAT2	↑	Tissue	Diagnostic/Prognostic	(1, 18)
XIST	↑	Tissue/Cell lines	Prognostic/Therapeutic	(19, 20)
PANDAR	↑	Tissue/Cell lines	Prognostic	(21, 22)
H19	↑	Tissue/Cell lines	Diagnostic/Prognostic/Therapeutic	(23, 24)
SNHG6	↑	Tissue/Cell lines	Prognostic/Therapeutic	(25, 26)
LINC01510	↑	Tissue/Cell lines	Prognostic	(27, 28)
MIR4435-2HG	↑	Tissue	Diagnostic/Prognostic	(29, 30)
SLCO4A1-AS1	↑	Tissue/Cell lines	Prognostic/Therapeutic	(31, 32)
RP11-59H7.3	↑	Tissue/Serum/Cell lines	Diagnostic/Therapeutic	(33)
GACAT3	↑	Tissue	Diagnostic/Prognostic	(34)
LINC00152	↑	Tissue	Diagnostic/Prognostic	(34)
TRERNA1	↑	Tissue/Cell lines	Prognostic	(35)
LEF1-AS1	↑	Tissue/Cell lines	Therapeutic/Prognostic	(36)
B3GALT5-AS1	↓	Serum	Diagnostic	(37)
DANCR	↑	Serum	Diagnostic	(38)
HANR	↑	Tissue	Diagnostic/Prognostic	(39)
MFI2-AS1	↑	Tissue	Prognostic/Therapeutic	(40)
treRNA	↑	Tissue	Prognostic	(41)
LINC00461	↑	Tissue/Cell lines	Prognostic/Therapeutic	(42)
AP003555.2	↑	Tissue	Prognostic	(43)
AP006284.1	↑	Tissue	Prognostic	(43)
LINC01602	↑	Tissue	Prognostic	(43)
LINRIS	↑	Tissue/Cell lines	Prognostic/Therapeutic	(44)
cCSC1	↑	Tissue/Cell lines	Prognostic/Therapeutic	(45)
LINC01234	↑	Tissue	Prognostic	(46)
SNHG11	↑	Plasma	Diagnostic/Prognostic	(47)
AK001058	↑	Cell lines	Diagnostic/Therapeutic	(48)
DNAH17-AS1	↑	Tissue	Prognostic/Therapeutic	(49)
RP11-400N13.2	↑	Tissue	Prognostic/Therapeutic	(49)
LINC00957	↑	Tissue/Cell lines	Prognostic/Therapeutic	(50)
NKILA	↓	Tissue/Cell lines	Diagnostic/Prognostic	(51)
MEG3	↓	Tissue/Cell lines/Serum	Diagnostic/Prognostic	(52)
XIRP2-AS1	↓	Tissue/Cell lines	Prognostic/Therapeutic	(53)
CTA-941F9.9	↓	Tissue	Diagnostic	(54)
ZFAS1	↑	Tissue/Cell lines	Diagnostic/Therapeutic	(55)
LNRRIL6	↑	Tissue/Cell lines	Prognostic/Therapeutic	(56)
KIAA0125	↓	Tissue/Cell lines	Diagnostic	(57)
IQCJ-SCHIP1	↓	Tissue	Prognostic/Therapeutic	(58)
DILC	↓	Tissue	Diagnostic/Prognostic	(59)
CRCAL-3	↑	Tissue/Cell lines	Diagnostic/Prognostic/Therapeutic	(60)
CASC19	↑	Tissue/Cell lines	Diagnostic/Therapeutic	(61)
HOTTIP	↑	Tissue	Diagnostic	(11)
PVT1	↑	Tissue	Diagnostic	(11)
UCA1	↑	Tissue	Diagnostic	(11)
RP11	↑	Tissue	Therapeutic	(62)
KAT7	↓	Tissue/Cell lines	Diagnostic/Therapeutic	(63)
TINCR	↑	Tissue/Cell lines	Diagnostic/Prognostic	(64)
GIHCG	↑	Tissue/Cell lines	Prognostic/Therapeutic	(65)
LUCAT1	↑	Tissue/Cell lines	Prognostic/Therapeutic	(66)
ENST00000547547	↓	Tissue/Cell lines	Prognostic/Diagnostic	(67)
MLK7–AS1	↑	Tissue/Cell lines	Diagnostic/Therapeutic	(68)
MAPKAPK5-AS1	↑	Tissue/Cell lines	Prognostic/Therapeutic	(69)
SNHG15	↑	Tissue	Prognostic	(70)
RP1–85F18.6	↑	Tissue/Cell lines	Diagnostic/Prognostic	(71)
NONHSAT074176.2	↓	Tissue	Diagnostic/Therapeutic	(72)
DLEU1	↑	Tissue/Cell lines	Therapeutic	(73)
CYTOR	↑	Tissue	Prognostic/Therapeutic	(74)
SPINT1-AS1	↑	Tissue/Serum exosomes	Prognostic/Therapeutic	(75)
u50535	↑	Tissue/Cell lines	Diagnostic/Prognostic	(76)
RP11-909B2.1	↓	Tissue/Cell lines	Diagnostic/Prognostic	(77)
AK098783	↑	Tissue	Prognostic	(78)
XLOC_010588	↑	Tissue/Cell lines	Diagnostic/Prognostic/Therapeutic	(79)
91H	↑	Serum exosomes	Diagnostic/Prognostic/Therapeutic	(80)
SNHG1	↑	Tissue/Cell lines	Diagnostic/Therapeutic	(81)
LINC00959	↓	Tissue/Cell lines	Prognostic	(82)
GHRLOS	↓	Tissue	Prognostic/Therapeutic	(83)
CPS1-IT1	↓	Tissue/Cell lines	Prognostic/Therapeutic	(84)
HNF1A-AS1	↑	Tissue/Cell lines	Prognostic/Therapeutic	(85)
HOXA-AS2	↑	Tissue/Cell lines	Diagnostic/Therapeutic	(86)
HEIH	↑	Tissue/Cell lines	Prognostic/Therapeutic	(87)
NONHSAT062994	↓	Tissue/Cell lines	Prognostic/Therapeutic	(88)
BANCR	↑	Tissue	Prognostic	(89)
LL22NC03-N64E9.1	↑	Tissue/Cell lines	Prognostic/Therapeutic	(90)
ZEB1-AS1	↑	Tissue/Cell lines	Prognostic	(20)
BCAT1	↓	Tissue/Cell lines	Prognostic	(91)
BLACAT1	↑	Tissue/Cell lines	Diagnostic/Therapeutic	(92)
UBC1	↑	Tissue/Cell lines	Diagnostic	(93)
SNHG12	↑	Tissue/Cell lines	Diagnostic/Prognostic	(94)
SPRY4-IT1	↑	Tissue	Prognostic	(95)
HOXA11-AS	↓	Tissue/Cell lines	Prognostic/Therapeutic	(96)
CRNDE-h	↑	Serum exosomes	Diagnostic/Prognostic	(97)
Loc554202	↓	Tissue	Prognostic	(98)
FOXP4-AS1	↑	Tissue/Cell lines	Prognostic/Therapeutic	(99)
HOTAIRM1	↓	Tissue/Cell lines	Diagnostic	(100)
CTNNAP1	↓	Tissue	Diagnostic	(101)
LINC01133	↓	Tissue/Cell lines	Prognostic/Therapeutic	(102)
CASC11	↑	Tissue/Cell lines	Diagnostic/Therapeutic	(103)
TUG1	↑	Tissue/Cell lines	Diagnostic/Prognostic/Therapeutic	(104)
PRNCR1	↑	Tissue/Cell lines	Diagnostic	(105)
ATB	↑	Plasma	Diagnostic/Prognostic	(2)

References showed in **Supplementary Material 2**.

CRC-related transcription-1 (CCAT1) is a newly discovered lncRNA with a length of 2628 nucleotides (70). CCAT1 expression is upregulated in CRC (71), with CCAT1 levels on average 235 times higher in CRC tissue than in normal mucosa. In CRC patients, CCAT1 overexpression is detected in all hematoxylin and eosin-positive lymph nodes, and its detection rate in hematoxylin and eosin- and immunohistochemical-negative lymph nodes reaches 40.0%. CCAT1 is also highly expressed in peripheral blood samples from CRC patients. High CCAT1 expression indicates that CCAT1 can be utilized for the screening, diagnosis, and assessment of staging and overall prognosis of CRC patients (71). CCAT1 expression is significant in the progression of colonic adenoma to cancer, suggesting that it plays an important role in tumor genesis and metastasis (72). The combination of CCAT1 with another lncRNA (HOTAIR) provides higher diagnostic performance (73). CCAT1 alone or in combination with CCAT2 can be utilized as an important prognostic biomarker in CRC (74).

lncRNAs and miRNAs play mutual regulation roles, acting as competitive endogenous RNAs (ceRNAs) (75). Highly expressed CCAT1 targets and regulates miR-181a-5p, and is negatively correlated with its expression. CCAT1 and miR-181a-5p may act as ceRNAs, which can affect the growth of CRC tumors by regulating the p53 signaling pathway (76). The upregulation of CCAT1 expression decreases sensitivity to fluorouracil chemotherapy, whereas its downregulation effectively reverses the resistance of colon cancer cell lines to fluorouracil, thereby opening up a new approach for the treatment of colon cancer (77).

Metastatic lung adenocarcinoma transcript 1 (MALAT1), also known as nuclear-enriched transcript 2, is a highly conserved nuclear-enriched lncRNA of ~8,000 nucleotides, which was the first marker for the independent prognosis of early non-small cell lung cancer (78, 79). MALAT1 is upregulated in lung, breast, pancreatic, liver, prostate cancer and CRC, suggesting that it plays an important role in the pathogenesis and progression of cancer (80). MALAT1 can promote the growth and migration of CRC cells by competitively binding to the splicing factor proline- and glutamine-rich (SFPQ) tumor suppressor gene and releasing SFPQ from the SFPQ/polypyrimidine tract-binding protein 2 (PTBP2) complex, resulting in an increase in free proto-oncogene PTBP2, suggesting that MALAT1 can be a potential therapeutic target for CRC (81). MALAT1 binds to miR-15, inhibits the regulation of LDL receptor-related protein 6 expression by miR-15, and enhances β-catenin signaling, resulting in the downregulation of RUNX family transcription factor 2 (RUNX2) gene expression. Secondly, MALAT1 binds to SFPQ and dissociates SFPQ/PTBP2 dimers to release PTBP2, thereby increasing the translation of RUNX2 by interacting with the IRES domain in the 5′-untranslated region (UTR) of RUNX2 mRNA (82). The prognosis of patients with CRC tumors with high MALAT1 expression is significantly worse than those with low expression, suggesting that high MALAT1 expression may be a negative prognostic marker for patients with stage II/III CRC (83).

The lncRNA H19 is a maternally expressed imprinted gene that plays an important role in mammalian development (84, 85). H19 is substantially upregulated in CRC and has a carcinogenic effect (86). H19 combines with eukaryotic translation initiation factor 4A3 (eIF4A3) to prevent the recruitment of eIF4A3 to mRNAs encoding cell cycle genes, and then influences the expression of cell cycle regulation genes at the translation or post-translation level. High H19 expression in CRC is significantly correlated with the degree of tumor differentiation and advanced TNM stage, indicating its potential as a prognostic biomarker (87). Four selected single nucleotide polymorphisms in H19 (RS2839698, RS3024270, RS217727, and RS2735971) were genotyped and evaluated for their association with CRC risk in the Chinese population. The results showed that RS2839698 is associated with increased CRC risk, indicating its potential as a biomarker for predicting CRC susceptibility (88). The H19/miR-29b-3p/granulin precursor axis promotes epithelial-mesenchymal transition in CRC cells by acting on the Wnt/β-catenin signaling pathway, which suggests a direction for targeted gene therapy for CRC (89).

Pvt1 oncogene (PVT1) is an lncRNA that is greater than 30 kb in size (90) and is upregulated in cancers (especially various cancers of the digestive system, including esophageal, gastric, primary liver, and pancreatic cancer and CRC) and can promote tumor cell proliferation, migration, and invasion (91). PVT1 upregulation is usually associated with poor prognosis. High PVT1 expression helps to predict early metastasis or recurrence of CRC after radical resection, and is a prospective prognostic marker (92). PVT1 overexpression may promote multidrug resistance in CRC cells, and PVT1 knockdown can reverse the resistance of CRC cells to fluorouracil. These observations indicate that PVT1 is a potential target for the treatment of multidrug resistance in CRC (93). The diagnostic sensitivity and specificity of PVT1 in CRC patients are 72.5% and 87.5%, respectively, and the AUC value is 0.856. It has high diagnostic performance, indicating that it has good clinical value for the early diagnosis of CRC (94).

### circRNAs as Biomarkers for the Diagnosis and Prognosis of CRC

circRNAs are endogenous molecules formed by the reverse splicing of exons, introns, or both exons and introns, resulting in exonic or intronic circRNAs (95). Compared with their linear counterparts, they are highly stable, abundant, and evolutionarily conserved, indicating that they may have important regulatory roles in the development of human tumors (96). circRNAs have a covalently closed loop structure with no 5′-cap or 3′-polyadenylic acid tail, and they are not sensitive to digestion by RNase enzymes (97). Due to the stability of circRNAs, they are abundant in the cytoplasm, and their cellular level can be adjusted by exosome removal or core activity (98).

Although the biological functions of circRNAs are largely not known, previous reports have confirmed that their functions can be divided into roughly four aspects: acting as miRNA sponges, interacting with RNA-binding proteins, encoding proteins, and regulating transcription (99). First, the most researched circRNA molecules are effective miRNA sponges for the regulation of gene expression (100–103). circRNAs may act as miRNA sponges through ceRNA networks, leading to the upregulation or downregulation of target miRNA expression. A single circRNA can bind one or more miRNAs, affecting the translation of dozens or even hundreds of ceRNA transcripts (104). Second, circRNAs have been described as “scaffolds” that interact with many RNA‐binding proteins to regulate gene expression. Third, some circRNAs can code for proteins. SHPRH‐146aa is a new protein produced by the SNF2 histone linker PHD helicase (SHPRH) gene. The SHPRH circRNA uses overlapping genetic codes to produce a UGA termination codon, resulting in the translation of 17-kDa SHPRH‐146aa (105). Fourth, circRNAs may regulate transcription. Studies have shown that circ-ANKRD52 is a circRNA derived from an intron in ankyrin repeat domain 52 (ANKRD52). The combination of circ-ANKRD52 and RNA Pol II knockdown reduces the expression of the parental genes, suggesting that circRNAs may be positive regulators of RNA Pol II transcription (106). circRNAs have a wide range of expression patterns and have unique characteristics such as tissue specificity, stability, and evolutionary conservation, so they may become ideal biomarkers (107). Importantly, circRNAs are stably expressed in saliva, blood, and exosomes, which further increases their potential as biomarkers for disease diagnosis and prognosis (108).

In the past 5 years, with the development of high-throughput sequencing technology, studies of circRNAs as CRC biomarkers have become ever more extensive. circRNAs are differentially expressed in many cancers, including CRC. The expression of hsa_circ_001988 is significantly downregulated in CRC tissue. The sensitivity and specificity of hsa_circ_001988 for diagnosing CRC are 68% and 73%, respectively, with an AUC value of 0.788. These results indicate that hsa_circ_001988 is a potential biomarker for the diagnosis of CRC (109). circLMNB1, encoded by lamin B1 (LMNB1), is highly expressed in CRC tissue and in 5 CRC cell lines (HT29, LoVo, HCT116, SW480, and RKO). Knockout of circLMNB1 upregulates the expression of E-cadherin, Bax, and caspase-3 in LoVo cells and downregulates the expression of matrix metallopeptidase 2, matrix metallopeptidase 9, and N-cadherin to inhibit the proliferation, migration, and invasion of LoVo cells and to promote cell cycle arrest and apoptosis, indicating that circLMNB1 can be a potential therapeutic target for CRC patients (110). circHIPK3 (hsa_circ_0000284) is derived from exon 2 of the homeodomain-interacting protein kinase 3 (HIPK3) gene, and its spliced mature sequence length is 1099 nucleotides. circHIPK3 is also highly expressed in CRC tissues (111). Elevated circHIPK3 expression is an independent prognostic factor for low overall survival in CRC, which means that circHIPK3 may be a promising prognostic biomarker in CRC. circHIPK3 acts as a miRNA sponge for miR-7 in CRC. Overexpression of circHIPK3 effectively reverses miR-7-induced inhibition of CRC cells progression, and circHIPK3 is regulated by the upstream transcription factor c-Myb. The signaling pathway formed by the c-Myb/circHIPK3/miR-7 axis can also be used as a potential target for the treatment of CRC (111).

Research on circRNAs is still in its infancy, and there are still many challenges to be faced. The mechanisms by which circRNAs participate in the progression of CRC are very complicated. Studies that have determined the partial functions of circRNAs in CRC are shown in **Table 3**. However, there are more circRNAs for CRC that need to be studied. Their functions, mechanisms of action, and clinical application need to be further clarified.

**Table 3:** circRNAs as biomarkers for the diagnosis and prognosis of CRC.

CircRNAs	Targeted miRNA	Regulatory Role of circRNA on miRNA	The expression of circRNA	Potential biomarkers	Reference
circSMARCC1	miR-140-3p	Negative	↑	Therapeutic	(1)
	hsa-miR-6833-				(2)
circ-PNN	3p/hsa-let-7i-3p/hsa-miR-1301-3p	Negative	↑	Diagnostic	
circRNA_101951	/	/	↑	Prognostic/Therapeutic	(3)
circPTK2	/	/	↑	Diagnostic/Therapeutic	(4)
circCAMSAP1	miR-328-5p	Negative	↑	Prognostic/Diagnostic/Therapeutic	(5)
circ-0004771	/	/	↑	Diagnostic	(6)
circ_0000338	/	/	↑	Therapeutic	(7)
hsa_circ_0082182			↑		
				Diagnostic	(8)
hsa_circ_0000370 hsa_circ_0035445	/	/	↑↓		
circ-CCDC66			↓		(9)
circ-ABCC1 circ-STIL	/	/	↓↓	Diagnostic	
circ-ITGA7	miR-3187-3p	Negative	↓	Diagnostic/Therapeutic	(10)
hsa_circ_0004585	Multiple	/	↑	Diagnostic/Therapeutic	(11)
hsa_circ_0142527	/	/	↓	Diagnostic	(12)
circVAPA	miR-101	Negative	↑	Diagnostic/Therapeutic	(13)
circDDX17	hsa-miR-21-5p	Negative	↓	Diagnostic/Therapeutic	(14)
circ_0026344	miR-21/miR-31	Negative	↓	Prognostic	(15)
circHIPK3	miR-7	Negative	↑	Prognostic/Therapeutic	(16)
hsa_circ_0007534	/	/	↑	Diagnostic/Prognostic	(17)
circRNA0003906	/	/	↓	Diagnostic/Therapeutic	(18)
hsa_circRNA_103809	/	/	↓	Diagnostic	(19)
hsa_circRNA_104700			↓		
circRNA_001569	miR-145	Negative	↑	Therapeutic	(20)
hsa_circ_0005075	/	/	↑	Diagnostic/Therapeutic	(21)
hsa_circ_0020397	miR-138	Negative	↑	Therapeutic	(22)
hsa_circ_0136666	miR-136	Negative	↑	Therapeutic	(23)
hsa_circ_0000523	miR-31	Negative	↓	Therapeutic	(24)
has_circ_0055625	miR‐106b‐5p	Negative	↑	Therapeutic/Prognostic	(25)
circ_104916	/	/	↓	Therapeutic/Prognostic	(26)
hsa_circ_0000423	/	/	↑	Therapeutic/Prognostic	(27)
hsa_circ_0001649	/	/	↓	Diagnostic	(28)
hsa_circ_0000567	/	/	↓	Diagnostic/Prognostic	(29)
hsa_circ_0000826	/	/	↓	Diagnostic/Therapeutic	(30)
circ-FBXW7	/	/	↓	Therapeutic	(31)
circMTO1	/	/	↓	Therapeutic/Prognostic	(32)
circ_0002138	/	/	↓	Diagnostic/Therapeutic	(33)
hsa_circ_0002320	/	/	↓	Diagnostic/Prognostic	(34)
hsa_circ_0000711	/	/	↓	Diagnostic/Prognostic	(35)
circLMNB1	/	/	↑	Therapeutic	(36)
circFADS2	/	/	↑	Therapeutic/Prognostic	(37)
circUBAP2	miR-199a	Negative	↑	Therapeutic	(38)
hsa_circ_0044556	hsa-mir-214-3p	Negative	↑	Diagnostic/Therapeutic/Prognostic	(39)
circVAPA	miR-125a	Negative	↑	Therapeutic	(40)
circHUWE1	miR-486	Negative	↑	Diagnostic/Therapeutic	(41)
circMBOAT2	miR-519d-3p	Negative	↑	Diagnostic/Prognostic	(42)

References showed in **Supplementary Material 3**.

### Small Nucleolar RNAs (snoRNAs) as Biomarkers for the Diagnosis and Prognosis of CRC

snoRNAs are a type of generally recognized ncRNA molecule with a length of 60-300 nucleotides that are located mainly in the nucleolus (112, 113). There are two major types of snoRNAs,namely, C/D box snoRNAs and H/ACA box snoRNAs. They differ in their sequence, structure, binding partners, and the nature of post-transcriptional modifications they induce (114). Traditionally, snoRNAs have been considered housekeeping genes because they promote the modification, maturation, and stabilization of pre-ribosomal RNAs by inducing 2′-o-methylation or pseudo-nuclear modifications at specific pre-ribosomal RNA sites with the help of small nucleolar ribonucleoproteins (115). However, more recently, there has been some evidence that they have carcinogenic or anticancer roles (116–118). snoRNAs exist in a stable form in plasma, sputum, and urine samples (118); therefore, they have the potential to be fluid-based biomarkers for cancer (**Table 4**).

**Table 4:** snoRNA as potential biomarker for colorectal cancer.

Cancer	snoRNA	Expression	Sample	Potential biomarker	Function of RNA	Reference
Colorectal cancer	SNORA42	↑	Tissue/Cell lines	Prognostic	Oncogene	(1)
Colorectal cancer	SNORA21	↑	Tissue/Cell lines	Diagnostic/Prognostic/Therapeutic	Oncogene	(2)
Hepatocellular carcinoma/Colorectal cancer	SNORD126	↑	Tissue	Therapeutic	Oncogene	(3)
Hepatocellular carcinoma	SNORACA11	↑	Tissue/Cell lines	Prognostic/Therapeutic	Oncogene	(4)
Lung carcinoma	SNORA71A	↑	Tissue	Prognostic/Therapeutic	Oncogene	(5)
Gastric carcinoma	SNORD105b	↑	Tissue	Prognostic/Therapeutic	Oncogene	(6)
Breast carcinoma	SNORA7B	↑	Tissue/Cell lines	Diagnostic/Prognostic	Oncogene	(7)
Prostate cancer	SNORA42	↑	Tissue/Cell lines	Diagnostic/Prognostic	Oncogene	(8)

References showed in **Supplementary Material 4**.

The high SNORA42 expression is significantly correlated with a reduction of overall survival and disease-free survival in CRC patients, suggesting that high SNORA42 expression can be used as a prognostic biomarker for CRC (119). The expression of SNORA21 is significantly higher in adenoma and CRC tissues than in adjacent tissue. Receiver operating characteristic curve analysis showed that SNORA21 expression could distinguish CRC tissue from adjacent tissue and that SNORA21 could be used as a diagnostic biomarker for colorectal tumors. Elevated SNORA21 expression also significantly correlates with TNM staging and distant metastasis of CRC, and these results indicate that SNORA21 is also a putative prognostic biomarker for CRC (120). SNORD126 is remarkably highly expressed in tissue samples from CRC patients. SNORD126 promotes the growth of CRC cells by activating the PI3K-Akt pathway *via* the upregulation of fibroblast growth factor receptor 2 expression, and SNORD126 may be a potential therapeutic biomarker for CRC (121).

### Transfer RNAs (tRNAs), tRNA-Derived Fragments (tRFs), and tRNA Stress-Induced Small RNAs (tiRNAs) as Biomarkers for CRC

tRNAs are ncRNAs with a length of 76–90 nucleotides (122). tRNAs deliver amino acids to ribosomes and play a key role in protein synthesis (123). According to the length and cutting site of tRNAs, tRNA-derived small RNAs can be divided into two main types (1): tRFs, 14-30 nucleotides in length, derived from mature or precursor tRNAs; and (2) tiRNAs, 29-50 nucleotides in length, induced by stress and produced by specific cleavage of the anticodon loop of mature tRNAs (124). tRF/miR-1280 (derived from both pre-miRNA and tRNA-Leu) levels are significantly reduced in CRC tissue compared with adjacent tissue (125). tRF/miR-1280 is a fundamental regulator of cancer stem cell growth and function in CRC cells. tRF/miR-1280 inhibits Notch/GATA and miR-200b signal transduction through its direct interaction with the 3′-UTR of Jagged canonical Notch ligand 2 (125). According to whether they contain a 5′- or 3′-sequence, tiRNAs can be divided into two subtypes: 5′-tiRNAs and 3′-tiRNAs (126). The expression of 5′-tiRNA-Val is significantly higher in the serum of CRC patients compared with healthy controls, and the average relative level of 5′-tiRNA-Val is higher in CRC tissues with metastasis than in CRC tissues without metastasis, indicating that 5′-tiRNA-Val is a potential biomarker for assessing the progression of CRC (127). However, very little research has been conducted on the application of tRNAs, tRFs, and tiRNAs as CRC biomarkers.

### piRNAs as Biomarkers for CRC

piRNAs are a newly discovered class of small RNA molecules that are expressed mainly in germ cell lines and play important roles in maintaining the DNA integrity of germ lines, inhibiting transposon transcription, inhibiting translation, participating in the formation of heterochromatin, epigenetic regulation, and germ cell maturation (128, 129). In addition, some studies have shown that piRNAs regulate mRNA expression by binding to the 3′-UTR of mRNAs (130, 131). More than 30,000 piRNAs have been identified in humans (132) and are believed to be related to the biological behavior of cancer and participate in the occurrence and development of cancer (129). piRNAs have recently been shown to be potential prognostic biomarkers for CRC (133). The expression of piR-1245 is significantly higher in CRC tissue than in paracancerous tissue. piR-1245 is not only highly expressed in CRC tissue but is also upregulated in other types of cancer (including lung, breast, stomach, bladder, kidney, and prostate cancer), highlighting its important role in carcinogenesis. Meanwhile, high piR-1245 expression is an independent predictor of poor prognosis in CRC (133). The expression of 5 piRNAs (piR-001311, piR-004153, piR-017723, piR-017724, and piR-020365) is markedly downregulated in CRC patients (134). The AUC value of these 5 piRNAs is 0.867 (95%CI, 0.817-0.907), with a sensitivity of 78.3% and specificity of 74.2%. These findings suggest that these 5 serum piRNAs may be potential diagnostic biomarkers for CRC (134). Other putative piRNA biomarkers of CRC are shown in **Table 5**.

**Table 5:** piRNAs as potential biomarker for colorectal cancer.

piRNA	Expression	Sample	Biomarker	Reference
piR-1245	↑	Tissue	Prognostic	(1)
piR-001311piR-004153				
piR-017723piR-017724piR-020365	↓	Serum	Diagnostic/Prognostic	(2)
piR-823	↑	Tissue	Prognostic/Therapeutic	(3)
piR-020619piR-020450	↑	Serum	Diagnostic	(4)
piR-24000	↑	Tissue	Diagnostic/Therapeutic	(5)
piR-54265	↑	Tissue/Cell lines	Therapeutic/Prognostic	(6)

References showed in **Supplementary Material 5**.

## Conclusion and Future Outlook

**Figure 1:**
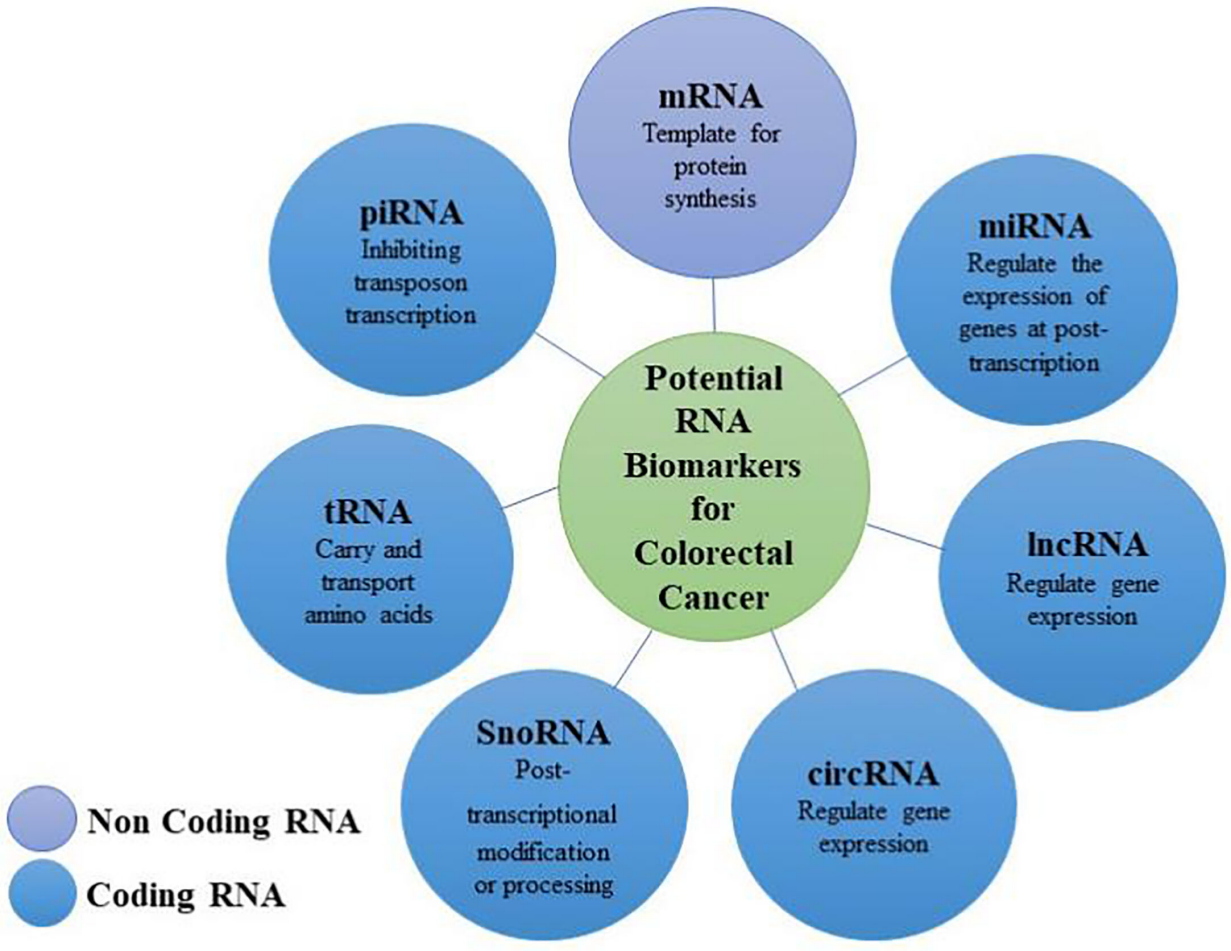
Biomarkers (mRNAs and non-coding RNAs) for the diagnosis and prognosis of colorectal cancer.

CRC is one of the most common malignant tumors in humans, with more than half a million deaths each year, and a delayed diagnosis is one of the most critical problems of CRC. Despite significant efforts and progress in improving the treatment of CRC through surgery and chemotherapy, its prognosis remains poor. In addition, recurrence and metastasis often occur after surgery. However, it is encouraging that screening has become routine in many countries, and newer, less invasive techniques are being developed to replace highly invasive colonoscopies. In these less invasive or non-invasive methods, further progress is needed to achieve early diagnosis, preoperative and postoperative staging, and to predict the clinical prognosis of CRC. Increasing evidence shows that ncRNAs play vital roles in the occurrence and development of CRC. The emergence of high-throughput sequencing technology and the study of epigenetics and transcriptomics have also further promoted our understanding of CRC. In this review, we described the performance of various RNAs as potential biomarkers for CRC, including the transition from tissue samples and cell line models to body fluid biopsies. In addition to the diagnostic performance and prognostic value of a single RNA biomarker, a variety of RNA biomarker combinations can improve the sensitivity and specificity of diagnosis and prognosis. Being able to detect RNAs in various body fluids is their main advantage as biomarkers so that a non-invasive diagnosis can be made. There are numerous studies on RNA biomarkers of CRC, but there are few unified opinions at present. The goal of current and future studies is to determine which non-invasive CRC diagnostic biomarkers are feasible, to understand which biomarkers can better predict patient prognosis, and to seek more personalized therapeutic targets (**Figure 1**).

## Author Contributions

JH and FW wrote the manuscript. All authors contributed to the article and approved the submitted version.

## Funding

This work was supported by grants from the Medical and Health Science and Technology Project of Panyu District, Guangzhou (No. 2017-Z04-18, 2018-Z04-59, 2018-Z04-50, 2019-Z04-02), Science and Technology Planning Project of Guangdong Province (No. 2017ZC0372), Guangzhou Health and Family Planning Commission Program (No. 20181A011118, 20192A011027, 20191A011119, 20201A010085), Project of Guangdong Administration of Traditional Chinese Medicine (No. 20192073), Natural Science Foundation of Guangdong Province (No. 2018A0303130191), Guangzhou Science and Technology Plan Project (No.201904010044, 202002030032), and Medical Science and Technology Research Foundation of Guangdong Province (No. A2020304).

## Conflict of Interest

The authors declare that the research was conducted in the absence of any commercial or financial relationships that could be construed as a potential conflict of interest.
