# Corrigendum: Biomarkers (mRNAs and non-coding RNAs) for the diagnosis and prognosis of colorectal cancer - from the body fluid to tissue level

**DOI:** 10.3389/fonc.2024.1476176

**Published:** 2024-09-13

**Authors:** Jinhua He, Feifeng Wu, Zeping Han, Min Hu, Weida Lin, Yuguang Li, Mingrong Cao

**Affiliations:** ^1^ Department of Laboratory Medicine, Central Hospital of Panyu District, Guangzhou, China; ^2^ Department of Hepatobiliary Surgery, The First Affiliated Hospital of Jinan University, Guangzhou, China

**Keywords:** colorectal cancer, circulating tumor cell, circulating tumor DNA, circulating tumor miRNA, exosomes

In the published article, there was an error. There is a clerical error in our article. On the page 5, the word “increase” should actually be “decrease”. So, it should read, “The upregulation of CCAT1 expression decreases sensitivity to fluorouracil chemotherapy, whereas its downregulation effectively reverses the resistance of colon cancer cell lines to fluorouracil, thereby opening up a new approach for the treatment of colon cancer.”

A correction has been made to **Candidate RNA Molecules as Biomarkers for CRC**.

*lncRNAs as Biomarkers for CRC*, Paragraph 3. This sentence previously stated:

“The upregulation of CCAT1 expression increases sensitivity to fluorouracil chemotherapy, whereas its downregulation effectively reverses the resistance of colon cancer cell lines to fluorouracil, thereby opening up a new approach for the treatment of colon cancer.”

The corrected sentence appears below:

“The upregulation of CCAT1 expression decreases sensitivity to fluorouracil chemotherapy, whereas its downregulation effectively reverses the resistance of colon cancer cell lines to fluorouracil, thereby opening up a new approach for the treatment of colon cancer.”

The authors apologize for this error and state that this does not change the scientific conclusions of the article in any way. The original article has been updated.

