# Supplementary material for: Biomarkers (mRNAs and Non-Coding RNAs) for the Diagnosis and Prognosis of Colorectal Cancer – From the Body Fluid to Tissue Level
Source: Front Oncol. 2021 Apr 29;11:632834. doi: 10.3389/fonc.2021.632834 (PMC8118670; doi:10.3389/fonc.2021.632834)
Supplement: Supplementary file 1 [file DataSheet_1.docx]

**Supplementary material 1**

**References(As showed in table 1, microRNAs as potential biomarkers for colorectal cancer)**

1. Liu Q, Yang W, Luo Y, Hu S, Zhu L. Correlation between miR-21 and miR-145 and the incidence and prognosis of colorectal cancer. *J BUON*(2018*)* 23*(*1)*:*29-35. doi:

2. Tsukamoto M, Iinuma H, Yagi T, Matsuda K, Hashiguchi Y. Circulating Exosomal MicroRNA-21 as a Biomarker in Each Tumor Stage of Colorectal Cancer. *Oncology*(2017*)* 92*(*6)*:*360-70. doi: 10.1159/000463387

3. Lai X, Friedman A. Exosomal microRNA concentrations in colorectal cancer: A mathematical model. *J Theor Biol*(2017*)* 415*:*70-83. doi: 10.1016/j.jtbi.2016.12.006

4. Uratani R, Toiyama Y, Kitajima T, Kawamura M, Hiro J, Kobayashi M, et al. Diagnostic Potential of Cell-Free and Exosomal MicroRNAs in the Identification of Patients with High-Risk Colorectal Adenomas. *PLoS One*(2016*)* 11*(*10)*:*e0160722. doi: 10.1371/journal.pone.0160722

5. Karimi N, Ali Hosseinpour Feizi M, Safaralizadeh R, Hashemzadeh S, Baradaran B, Shokouhi B, et al. Serum overexpression of miR-301a and miR-23a in patients with colorectal cancer. *J Chin Med Assoc*(2019*)* 82*(*3)*:*215-20. doi: 10.1097/JCMA.0000000000000031

6. Zhang H, Zhu M, Shan X, Zhou X, Wang T, Zhang J, et al. A panel of seven-miRNA signature in plasma as potential biomarker for colorectal cancer diagnosis. *Gene*(2019*)* 687*:*246-54. doi: 10.1016/j.gene.2018.11.055

7. Fu F, Jiang W, Zhou L, Chen Z. Circulating Exosomal miR-17-5p and miR-92a-3p Predict Pathologic Stage and Grade of Colorectal Cancer. *Transl Oncol*(2018*)* 11*(*2)*:*221-32. doi: 10.1016/j.tranon.2017.12.012

8. Zhao YJ, Song X, Niu L, Tang Y, Song X, Xie L. Circulating Exosomal miR-150-5p and miR-99b-5p as Diagnostic Biomarkers for Colorectal Cancer. *Front Oncol*(2019*)* 9*:*1129. doi: 10.3389/fonc.2019.01129

9. Zou SL, Chen YL, Ge ZZ, Qu YY, Cao Y, Kang ZX. Downregulation of serum exosomal miR-150-5p is associated with poor prognosis in patients with colorectal cancer. *Cancer Biomark*(2019*)* 26*(*1)*:*69-77. doi: 10.3233/CBM-190156

10. Zhu Y, Xu A, Li J, Fu J, Wang G, Yang Y, et al. Fecal miR-29a and miR-224 as the noninvasive biomarkers for colorectal cancer. *Cancer Biomark*(2016*)* 16*(*2)*:*259-64. doi: 10.3233/CBM-150563

11. Sun L, Liu X, Pan B, Hu X, Zhu Y, Su Y, et al. Serum exosomal miR-122 as a potential diagnostic and prognostic biomarker of colorectal cancer with liver metastasis. *J Cancer*(2020*)* 11*(*3)*:*630-7. doi: 10.7150/jca.33022

12. Baek DW, Kim G, Kang BW, Kim HJ, Park SY, Park JS, et al. High expression of microRNA-199a-5p is associated with superior clinical outcomes in patients with locally advanced rectal cancer. *J Cancer Res Clin Oncol*(2020*)* 146*(*1)*:*105-15. doi: 10.1007/s00432-019-03099-4

13. Chen HL, Li JJ, Jiang F, Shi WJ, Chang GY. MicroRNA-4461 derived from bone marrow mesenchymal stem cell exosomes inhibits tumorigenesis by downregulating COPB2 expression in colorectal cancer. *Biosci Biotechnol Biochem*(2020*)* 84*(*2)*:*338-46. doi: 10.1080/09168451.2019.1677452

14. Min L, Chen L, Liu S, Yu Y, Guo Q, Li P, et al. Loss of Circulating Exosomal miR-92b is a Novel Biomarker of Colorectal Cancer at Early Stage. *Int J Med Sci*(2019*)* 16*(*9)*:*1231-7. doi: 10.7150/ijms.34540

15. Tang Y, Zhao Y, Song X, Song X, Niu L, Xie L. Tumor-derived exosomal miRNA-320d as a biomarker for metastatic colorectal cancer. *J Clin Lab Anal*(2019*)* 33*(*9)*:*e23004. doi: 10.1002/jcla.23004

16. Peng ZY, Gu RH, Yan B. Downregulation of exosome-encapsulated miR-548c-5p is associated with poor prognosis in colorectal cancer. *J Cell Biochem*(2018*)* doi: 10.1002/jcb.27291

17. Liu X, Pan B, Sun L, Chen X, Zeng K, Hu X, et al. Circulating Exosomal miR-27a and miR-130a Act as Novel Diagnostic and Prognostic Biomarkers of Colorectal Cancer. *Cancer Epidemiol Biomarkers Prev*(2018*)* 27*(*7)*:*746-54. doi: 10.1158/1055-9965.EPI-18-0067

18. Yan S, Jiang Y, Liang C, Cheng M, Jin C, Duan Q, et al. Exosomal miR-6803-5p as potential diagnostic and prognostic marker in colorectal cancer. *J Cell Biochem*(2018*)* 119*(*5)*:*4113-9. doi: 10.1002/jcb.26609

19. Yan S, Liu G, Jin C, Wang Z, Duan Q, Xu J, et al. MicroRNA-6869-5p acts as a tumor suppressor via targeting TLR4/NF-kappaB signaling pathway in colorectal cancer. *J Cell Physiol*(2018*)* 233*(*9)*:*6660-8. doi: 10.1002/jcp.26316

20. Wang J, Yan F, Zhao Q, Zhan F, Wang R, Wang L, et al. Circulating exosomal miR-125a-3p as a novel biomarker for early-stage colon cancer. *Sci Rep*(2017*)* 7*(*1)*:*4150. doi: 10.1038/s41598-017-04386-1

21. Li J, Chen Y, Guo X, Zhou L, Jia Z, Peng Z, et al. GPC1 exosome and its regulatory miRNAs are specific markers for the detection and target therapy of colorectal cancer. *J Cell Mol Med*(2017*)* 21*(*5)*:*838-47. doi: 10.1111/jcmm.12941

22. Zhu Mx, Huang ZB, Zhu DB, Zhou X, Shan X, Qi LW, et al. A panel of microRNA signature in serum for colorectal cancer diagnosis. *Oncotarget*(2017*)* 8*(*10)*:*17081-91. doi: 10.18632/oncotarget.15059

23. Liu C, Eng C, Shen J, Lu Y, Takata Y, Mehdizadeh A, et al. Serum exosomal miR-4772-3p is a predictor of tumor recurrence in stage II and III colon cancer. *Oncotarget*(2016*)* 7*(*46)*:*76250-60. doi: 10.18632/oncotarget.12841

24. Matsumura T, Sugimachi K, Iinuma H, Takahashi Y, Kurashige J, Sawada G, et al. Exosomal microRNA in serum is a novel biomarker of recurrence in human colorectal cancer. *Br J Cancer*(2015*)* 113*(*2)*:*275-81. doi: 10.1038/bjc.2015.201

25. Gao J, Li N, Dong Y, Li S, Xu L, Li X, et al. miR-34a-5p suppresses colorectal cancer metastasis and predicts recurrence in patients with stage II/III colorectal cancer. *Oncogene*(2015*)* 34*(*31)*:*4142-52. doi: 10.1038/onc.2014.348

26. Mokutani Y, Uemura M, Munakata K, Okuzaki D, Haraguchi N, Takahashi H, et al. Down-Regulation of microRNA-132 is Associated with Poor Prognosis of Colorectal Cancer. *Ann Surg Oncol*(2016*)* 23*(*Suppl 5)*:*599-608. doi: 10.1245/s10434-016-5133-3

27. Shen Z, Wang B, Jiang K, Ye C-X, Cheng C, Yan Y, et al. Downregulation of miR-199b is associated with distant metastasis in colorectal cancer via activation of SIRT1 and inhibition of CREB/KISS1 signaling. *Oncotarget*(2016*)* 7*(*23)*:*35092-105. doi: 10.18632/oncotarget.9042

28. Sun M, Song H, Wang S, Zhang C, Zheng L, Chen F, et al. Integrated analysis identifies microRNA-195 as a suppressor of Hippo-YAP pathway in colorectal cancer. *J Hematol Oncol*(2017*)* 10*(*1)*:*79. doi: 10.1186/s13045-017-0445-8

29. Chen Q, Zhou L, Ye X, Tao M, Wu J. miR-145-5p suppresses proliferation, metastasis and EMT of colorectal cancer by targeting CDCA3. *Pathol Res Pract*(2020*)* 216*(*4)*:*152872. doi: 10.1016/j.prp.2020.152872
