# Supplementary material for: Biomarkers (mRNAs and Non-Coding RNAs) for the Diagnosis and Prognosis of Colorectal Cancer – From the Body Fluid to Tissue Level
Source: Front Oncol. 2021 Apr 29;11:632834. doi: 10.3389/fonc.2021.632834 (PMC8118670; doi:10.3389/fonc.2021.632834)
Supplement: Supplementary file 2 [file DataSheet_2.docx]

**Supplementary material 2**

**References (As showed in Table 2. Potential long non-coding RNAs as biomarkers for the diagnosis and prognosis of colorectal cancer)**

1. Ozawa T, Matsuyama T, Toiyama Y, Takahashi N, Ishikawa T, Uetake H, et al. CCAT1 and CCAT2 long noncoding RNAs, located within the 8q.24.21 'gene desert', serve as important prognostic biomarkers in colorectal cancer. Ann Oncol(2017) 28(8):1882-8. doi: 10.1093/annonc/mdx248

2. Abedini P, Fattahi A, Agah S, Talebi A, Beygi AH, Amini SM, et al. Expression analysis of circulating plasma long noncoding RNAs in colorectal cancer: The relevance of lncRNAs ATB and CCAT1 as potential clinical hallmarks. J Cell Physiol(2019) 234(12):22028-33. doi: 10.1002/jcp.28765

3. Thiele JA, Hosek P, Kralovcova E, Ostasov P, Liska V, Bruha J, et al. lncRNAs in Non-Malignant Tissue Have Prognostic Value in Colorectal Cancer. Int J Mol Sci(2018) 19(9)doi: 10.3390/ijms19092672

4. Shang A, Wang W, Gu C, Chen W, Lu W, Sun Z, et al. Long non-coding RNA CCAT1 promotes colorectal cancer progression by regulating miR-181a-5p expression. Aging (Albany NY)(2020) 12(9):8301-20. doi: 10.18632/aging.103139

5. Huang KB, Zhang SP, Zhu YJ, Guo CH, Yang M, Liu J, et al. Hotair mediates tumorigenesis through recruiting EZH2 in colorectal cancer. J Cell Biochem(2019) 120(4):6071-7. doi: 10.1002/jcb.27893

6. Xiao Z, Qu Z, Chen Z, Fang Z, Zhou K, Huang Z, et al. LncRNA HOTAIR is a Prognostic Biomarker for the Proliferation and Chemoresistance of Colorectal Cancer via MiR-203a-3p-Mediated Wnt/ss-Catenin Signaling Pathway. Cell Physiol Biochem(2018) 46(3):1275-85. doi: 10.1159/000489110

7. Lin K, Jiang H, Zhang LL, Jiang Y, Yang YX, Qiu GD, et al. Down-Regulated LncRNA-HOTAIR Suppressed Colorectal Cancer Cell Proliferation, Invasion, and Migration by Mediating p21. Dig Dis Sci(2018) 63(9):2320-31. doi: 10.1007/s10620-018-5127-z

8. Liu H, Li A, Sun Z, Zhang J, Xu H. Long non-coding RNA NEAT1 promotes colorectal cancer progression by regulating miR-205-5p/VEGFA axis. Hum Cell(2020) 33(2):386-96. doi: 10.1007/s13577-019-00301-0

9. Luo Y, Chen JJ, Lv Q, Qin J, Huang YZ, Yu MH, et al. Long non-coding RNA NEAT1 promotes colorectal cancer progression by competitively binding miR-34a with SIRT1 and enhancing the Wnt/beta-catenin signaling pathway. Cancer Lett(2019) 440-441:11-22. doi: 10.1016/j.canlet.2018.10.002

10. Peng W, Wang Z, Fan H. LncRNA NEAT1 Impacts Cell Proliferation and Apoptosis of Colorectal Cancer via Regulation of Akt Signaling. Pathol Oncol Res(2017) 23(3):651-6. doi: 10.1007/s12253-016-0172-4

11. Gharib E, Anaraki F, Baghdar K, Ghavidel P, Sadeghi H, Nasrabadi PN, et al. Investigating the diagnostic performance of HOTTIP, PVT1, and UCA1 long noncoding RNAs as a predictive panel for the screening of colorectal cancer patients with lymph node metastasis. J Cell Biochem(2019) 120(9):14780-90. doi: 10.1002/jcb.28739

12. Fan H, Zhu JH, Yao XQ. Long non-coding RNA PVT1 as a novel potential biomarker for predicting the prognosis of colorectal cancer. Int J Biol Markers(2018) 33(4):415-22. doi: 10.1177/1724600818777242

13. Pan X, Cheng R, Zhu X, Cai F, Zheng G, Li J, et al. Prognostic Significance and Diagnostic Value of Overexpressed lncRNA PVT1 in Colorectal Cancer. Clin Lab(2019) 65(12)doi: 10.7754/Clin.Lab.2019.190412

14. Ji Q, Cai G, Liu X, Zhang Y, Wang Y, Zhou L, et al. MALAT1 regulates the transcriptional and translational levels of proto-oncogene RUNX2 in colorectal cancer metastasis. Cell Death Dis(2019) 10(6):378. doi: 10.1038/s41419-019-1598-x

15. Yang YN, Zhang R, Du JW, Yuan HH, Li YJ, Wei XL, et al. Predictive role of UCA1-containing exosomes in cetuximab-resistant colorectal cancer. Cancer Cell Int(2018) 18:164. doi: 10.1186/s12935-018-0660-6

16. Yu JH, Chen Y. Clinical significance of lncRNA BCYRN1 in colorectal cancer and its role in cell metastasis. Eur Rev Med Pharmacol Sci(2019) 23(21):9371-8. doi: 10.26355/eurrev_201911_19430

17. Gu L, Lu L, Zhou D, Liu Z. Long Noncoding RNA BCYRN1 Promotes the Proliferation of Colorectal Cancer Cells via Up-Regulating NPR3 Expression. Cell Physiol Biochem(2018) 48(6):2337-49. doi: 10.1159/000492649

18. Zhang J, Jiang Y, Zhu J, Wu T, Ma J, Du C, et al. Overexpression of long non-coding RNA colon cancer-associated transcript 2 is associated with advanced tumor progression and poor prognosis in patients with colorectal cancer. Oncol Lett(2017) 14(6):6907-14. doi: 10.3892/ol.2017.7049

19. Zhang XT, Pan SX, Wang AH, Kong QY, Jiang KT, Yu ZB. Long Non-Coding RNA (lncRNA) X-Inactive Specific Transcript (XIST) Plays a Critical Role in Predicting Clinical Prognosis and Progression of Colorectal Cancer. Med Sci Monit(2019) 25:6429-35. doi: 10.12659/MSM.915329

20. Chen DL, Chen LZ, Lu YX, Zhang DS, Zeng ZL, Pan ZZ, et al. Long noncoding RNA XIST expedites metastasis and modulates epithelial-mesenchymal transition in colorectal cancer. Cell Death Dis(2017) 8(8):e3011. doi: 10.1038/cddis.2017.421

21. Li X, Wang F, Sun Y, Fan Q, Cui G. Expression of long non-coding RNA PANDAR and its prognostic value in colorectal cancer patients. Int J Biol Markers(2017) 32(2):e218-e23. doi: 10.5301/jbm.5000249

22. Lu M, Liu Z, Li B, Wang G, Li D, Zhu Y. The high expression of long non-coding RNA PANDAR indicates a poor prognosis for colorectal cancer and promotes metastasis by EMT pathway. J Cancer Res Clin Oncol(2017) 143(1):71-81. doi: 10.1007/s00432-016-2252-y

23. Han D, Gao X, Wang M, Qiao Y, Xu Y, Yang J, et al. Long noncoding RNA H19 indicates a poor prognosis of colorectal cancer and promotes tumor growth by recruiting and binding to eIF4A3. Oncotarget(2016) 7(16):22159-73. doi: 10.18632/oncotarget.8063

24. Ding D, Li C, Zhao T, Li D, Yang L, Zhang B. LncRNA H19/miR-29b-3p/PGRN Axis Promoted Epithelial-Mesenchymal Transition of Colorectal Cancer Cells by Acting on Wnt Signaling. Mol Cells(2018) 41(5):423-35. doi: 10.14348/molcells.2018.2258

25. Yu C, Sun J, Leng X, Yang J. Long noncoding RNA SNHG6 functions as a competing endogenous RNA by sponging miR-181a-5p to regulate E2F5 expression in colorectal cancer. Cancer Manag Res(2019) 11:611-24. doi: 10.2147/CMAR.S182719

26. Wang X, Lai Q, He J, Li Q, Ding J, Lan Z, et al. LncRNA SNHG6 promotes proliferation, invasion and migration in colorectal cancer cells by activating TGF-beta/Smad signaling pathway via targeting UPF1 and inducing EMT via regulation of ZEB1. Int J Med Sci(2019) 16(1):51-9. doi: 10.7150/ijms.27359

27. Zhou JF, Wang HG, Ma TH, Yan W, Wu SN, Shi YT, et al. Long noncoding RNA LINC01510 is highly expressed in colorectal cancer and predicts favorable prognosis. Eur Rev Med Pharmacol Sci(2018) 22(22):7710-5. doi: 10.26355/eurrev_201811_16392

28. Cen C, Li J, Liu J, Yang M, Zhang T, Zuo Y, et al. Long noncoding RNA LINC01510 promotes the growth of colorectal cancer cells by modulating MET expression. Cancer Cell Int(2018) 18:45. doi: 10.1186/s12935-018-0503-5

29. Shen MY, Zhou GR, Zhang ZY. LncRNA MIR4435-2HG contributes into colorectal cancer development and predicts poor prognosis. Eur Rev Med Pharmacol Sci(2020) 24(4):1771-7. doi: 10.26355/eurrev_202002_20354

30. Ouyang W, Ren L, Liu G, Chi X, Wei H. LncRNA MIR4435-2HG predicts poor prognosis in patients with colorectal cancer. PeerJ(2019) 7:e6683. doi: 10.7717/peerj.6683

31. Tang R, Chen J, Tang M, Liao Z, Zhou L, Jiang J, et al. LncRNA SLCO4A1-AS1 predicts poor prognosis and promotes proliferation and metastasis via the EGFR/MAPK pathway in colorectal cancer. Int J Biol Sci(2019) 15(13):2885-96. doi: 10.7150/ijbs.38041

32. Yu J, Han Z, Sun Z, Wang Y, Zheng M, Song C. LncRNA SLCO4A1-AS1 facilitates growth and metastasis of colorectal cancer through beta-catenin-dependent Wnt pathway. J Exp Clin Cancer Res(2018) 37(1):222. doi: 10.1186/s13046-018-0896-y

33. Zhu XJ, Luo C, Bu FQ, Lin K, Zhu ZM. Long non-coding RNA RP11-59H7.3 promotes cell proliferation and invasion metastasis in colorectal cancer by miR-139-5p/NOTCH1 axis. Aging (Albany NY)(2020) 12(12):11653-66. doi: 10.18632/aging.103331

34. Ye S, Lu Y, Ru Y, Wu X, Zhao M, Chen J, et al. LncRNAs GACAT3 and LINC00152 regulate each other through miR-103 and are associated with clinicopathological characteristics in colorectal cancer. J Clin Lab Anal(2020) 34(9):e23378. doi: 10.1002/jcla.23378

35. Wang W, Tang X, Qu H, He Q. Translation regulatory long non-coding RNA 1 represents a potential prognostic biomarker for colorectal cancer. Oncol Lett(2020) 19(6):4077-87. doi: 10.3892/ol.2020.11532

36. Cheng Y, Wu J, Qin B, Zou BC, Wang YH, Li Y. CREB1-induced lncRNA LEF1-AS1 contributes to colorectal cancer progression via the miR-489/DIAPH1 axis. Biochem Biophys Res Commun(2020) 526(3):678-84. doi: 10.1016/j.bbrc.2020.03.153

37. Ding Y, Feng W, Ge JK, Dai L, Liu TT, Hua XY, et al. Serum level of long noncoding RNA B3GALT5-AS1 as a diagnostic biomarker of colorectal cancer. Future Oncol(2020) 16(13):827-35. doi: 10.2217/fon-2019-0820

38. Shen X, Xue Y, Cong H, Wang X, Fan Z, Cui X, et al. Circulating lncRNA DANCR as a potential auxillary biomarker for the diagnosis and prognostic prediction of colorectal cancer. Biosci Rep(2020) 40(3)doi: 10.1042/BSR20191481

39. Xu M, Guo X, Wang RD, Zhang ZH, Jia YM, Sun X. Long non-coding RNA HANR as a biomarker for the diagnosis and prognosis of colorectal cancer. Medicine (Baltimore)(2020) 99(7):e19066. doi: 10.1097/MD.0000000000019066

40. Luo R, Song J, Zhang W, Ran L. Identification of MFI2-AS1, a Novel Pivotal lncRNA for Prognosis of Stage III/IV Colorectal Cancer. Dig Dis Sci(2020) doi: 10.1007/s10620-020-06064-1

41. Chen S, Shan T, Chen X, Yang WB, Wu T, Sun XL, et al. Association of treRNA with lymphatic metastasis and poor prognosis in colorectal cancer. Int J Clin Exp Pathol(2019) 12(5):1770-4. doi:

42. Yu H, Ma J, Chen J, Yang Y, Liang J, Liang Y. LncRNA LINC00461 Promotes Colorectal Cancer Progression via miRNA-323b-3p/NFIB Axis. Onco Targets Ther(2019) 12:11119-29. doi: 10.2147/OTT.S228798

43. Liu Y, Liu B, Jin G, Zhang J, Wang X, Feng Y, et al. An Integrated Three-Long Non-coding RNA Signature Predicts Prognosis in Colorectal Cancer Patients. Front Oncol(2019) 9:1269. doi: 10.3389/fonc.2019.01269

44. Wang Y, Lu JH, Wu QN, Jin Y, Wang DS, Chen YX, et al. LncRNA LINRIS stabilizes IGF2BP2 and promotes the aerobic glycolysis in colorectal cancer. Mol Cancer(2019) 18(1):174. doi: 10.1186/s12943-019-1105-0

45. Zhou H, Xiong Y, Peng L, Wang R, Zhang H, Fu Z. LncRNA-cCSC1 modulates cancer stem cell properties in colorectal cancer via activation of the Hedgehog signaling pathway. J Cell Biochem(2020) 121(3):2510-24. doi: 10.1002/jcb.29473

46. Jiang X, Zhu Q, Wu P, Zhou F, Chen J. Upregulated Long Noncoding RNA LINC01234 Predicts Unfavorable Prognosis for Colorectal Cancer and Negatively Correlates With KLF6 Expression. Ann Lab Med(2020) 40(2):155-63. doi: 10.3343/alm.2020.40.2.155

47. Xu W, Zhou G, Wang H, Liu Y, Chen B, Chen W, et al. Circulating lncRNA SNHG11 as a novel biomarker for early diagnosis and prognosis of colorectal cancer. Int J Cancer(2020) 146(10):2901-12. doi: 10.1002/ijc.32747

48. Zheng S, Lin F, Zhang M, Fu J, Ge X, Mu N. AK001058 promotes the proliferation and migration of colorectal cancer cells by regulating methylation of ADAMTS12. Am J Transl Res(2019) 11(9):5869-78. doi:

49. Zhou W, Pan B, Liu L. Integrated bioinformatics analysis revealing independent prognostic long non-coding RNAs DNAH17-AS1 and RP11-400N13.2 and their potential oncogenic roles in colorectal cancer. Oncol Lett(2019) 18(4):3705-15. doi: 10.3892/ol.2019.10730

50. Zhang LH, Li LH, Zhang PF, Cai YF, Hua D. LINC00957 Acted as Prognostic Marker Was Associated With Fluorouracil Resistance in Human Colorectal Cancer. Front Oncol(2019) 9:776. doi: 10.3389/fonc.2019.00776

51. Jiang P, Han X, Zheng Y, Sui J, Bi W. Long non-coding RNA NKILA serves as a biomarker in the early diagnosis and prognosis of patients with colorectal cancer. Oncol Lett(2019) 18(2):2109-17. doi: 10.3892/ol.2019.10524

52. Wang W, Xie Y, Chen F, Liu X, Zhong LL, Wang HQ, et al. LncRNA MEG3 acts a biomarker and regulates cell functions by targeting ADAR1 in colorectal cancer. World J Gastroenterol(2019) 25(29):3972-84. doi: 10.3748/wjg.v25.i29.3972

53. Zhou F, Shen F, Zheng Z, Ruan J. The LncRNA XIRP2-AS1 predicts favorable prognosis in colon cancer. Onco Targets Ther(2019) 12:5767-78. doi: 10.2147/OTT.S215419

54. Guo Z, Zhou C, Zhong X, Shi J, Wu Z, Tang K, et al. The long noncoding RNA CTA-941F9.9 is frequently downregulated and may serve as a biomarker for carcinogenesis in colorectal cancer. J Clin Lab Anal(2019) 33(9):e22986. doi: 10.1002/jcla.22986

55. Mo D, Liu W, Li Y, Cui W. Long Non-coding RNA Zinc Finger Antisense 1 (ZFAS1) Regulates Proliferation, Migration, Invasion, and Apoptosis by Targeting MiR-7-5p in Colorectal Cancer. Med Sci Monit(2019) 25:5150-8. doi: 10.12659/MSM.916619

56. Wang J, Zhou J, Jiang C, Zheng J, Namba H, Chi P, et al. LNRRIL6, a novel long noncoding RNA, protects colorectal cancer cells by activating the IL-6-STAT3 pathway. Mol Oncol(2019) 13(11):2344-60. doi: 10.1002/1878-0261.12538

57. Yang Y, Zhao Y, Hu N, Zhao J, Bai Y. lncRNA KIAA0125 functions as a tumor suppressor modulating growth and metastasis of colorectal cancer via Wnt/beta-catenin pathway. Cell Biol Int(2019) doi: 10.1002/cbin.11196

58. Zhang J, Bian Z, Jin G, Liu Y, Li M, Yao S, et al. Long non-coding RNA IQCJ-SCHIP1 antisense RNA 1 is downregulated in colorectal cancer and inhibits cell proliferation. Ann Transl Med(2019) 7(9):198. doi: 10.21037/atm.2019.04.21

59. Li QG, Xu XQ, Zhou DY, Jia ZB, Yu BF, Xu FG, et al. Long non-coding RNA DILC as a potentially useful biomarker for the diagnosis and prognosis of colorectal cancer. Eur Rev Med Pharmacol Sci(2019) 23(8):3320-5. doi: 10.26355/eurrev_201904_17694

60. Chang H, Wang GN, Tao YL. The expression of long noncoding RNA CRCAL-3 in colorectal cancer and its impacts on cell proliferation and migration. J Cell Biochem(2019) 120(9):15369-77. doi: 10.1002/jcb.28804

61. Wang XD, Lu J, Lin YS, Gao C, Qi F. Functional role of long non-coding RNA CASC19/miR-140-5p/CEMIP axis in colorectal cancer progression in vitro. World J Gastroenterol(2019) 25(14):1697-714. doi: 10.3748/wjg.v25.i14.1697

62. Wu Y, Yang X, Chen Z, Tian L, Jiang G, Chen F, et al. m(6)A-induced lncRNA RP11 triggers the dissemination of colorectal cancer cells via upregulation of Zeb1. Mol Cancer(2019) 18(1):87. doi: 10.1186/s12943-019-1014-2

63. Wang Q, He R, Tan T, Li J, Hu Z, Luo W, et al. A novel long non-coding RNA-KAT7 is low expressed in colorectal cancer and acts as a tumor suppressor. Cancer Cell Int(2019) 19:40. doi: 10.1186/s12935-019-0760-y

64. Yu SJ, Wang D, Shao YK, Zhang T, Xie HT, Jiang XM, et al. SP1-induced lncRNA TINCR overexpression contributes to colorectal cancer progression by sponging miR-7-5p. Aging (Albany NY)(2019) 11(5):1389-403. doi: 10.18632/aging.101839

65. Jiang X, Li Q, Zhang S, Song C, Zheng P. Long noncoding RNA GIHCG induces cancer progression and chemoresistance and indicates poor prognosis in colorectal cancer. Onco Targets Ther(2019) 12:1059-70. doi: 10.2147/OTT.S192290

66. Zhou Q, Hou Z, Zuo S, Zhou X, Feng Y, Sun Y, et al. LUCAT1 promotes colorectal cancer tumorigenesis by targeting the ribosomal protein L40-MDM2-p53 pathway through binding with UBA52. Cancer Sci(2019) 110(4):1194-207. doi: 10.1111/cas.13951

67. Ai X, Lei Q, Shen X, Li J, Cen C, Xie G, et al. Long noncoding RNA ENST00000547547 inhibits cell proliferation, invasion and migration in colorectal cancer cells. Oncol Rep(2019) 41(1):483-91. doi: 10.3892/or.2018.6834

68. Zhang R, Li J, Yan X, Jin K, Li W, Liu X, et al. Long noncoding RNA MLK7AS1 promotes proliferation in human colorectal cancer via downregulation of p21 expression. Mol Med Rep(2019) 19(2):1210-21. doi: 10.3892/mmr.2018.9702

69. Ji H, Hui B, Wang J, Zhu Y, Tang L, Peng P, et al. Long noncoding RNA MAPKAPK5-AS1 promotes colorectal cancer proliferation by partly silencing p21 expression. Cancer Sci(2019) 110(1):72-85. doi: 10.1111/cas.13838

70. Huang L, Lin H, Kang L, Huang P, Huang J, Cai J, et al. Aberrant expression of long noncoding RNA SNHG15 correlates with liver metastasis and poor survival in colorectal cancer. J Cell Physiol(2019) 234(5):7032-9. doi: 10.1002/jcp.27456

71. Ma Y, Chen Y, Lin C, Hu G. Biological functions and clinical significance of the newly identified long noncoding RNA RP185F18.6 in colorectal cancer. Oncol Rep(2018) 40(5):2648-58. doi: 10.3892/or.2018.6694

72. Zhang Z, Jia H, Gu T, Hu Q, Yu J, Zang D, et al. RNA sequencing and bioinformatics analysis of the long noncoding RNA-mRNA network in colorectal cancer. J Cell Biochem(2018) 119(12):9957-66. doi: 10.1002/jcb.27319

73. Liu T, Han Z, Li H, Zhu Y, Sun Z, Zhu A. LncRNA DLEU1 contributes to colorectal cancer progression via activation of KPNA3. Mol Cancer(2018) 17(1):118. doi: 10.1186/s12943-018-0873-2

74. Wang X, Yu H, Sun W, Kong J, Zhang L, Tang J, et al. The long non-coding RNA CYTOR drives colorectal cancer progression by interacting with NCL and Sam68. Mol Cancer(2018) 17(1):110. doi: 10.1186/s12943-018-0860-7

75. Li C, Li W, Zhang Y, Zhang X, Liu T, Zhang Y, et al. Increased expression of antisense lncRNA SPINT1-AS1 predicts a poor prognosis in colorectal cancer and is negatively correlated with its sense transcript. Onco Targets Ther(2018) 11:3969-78. doi: 10.2147/OTT.S163883

76. Yu X, Yuan Z, Yang Z, Chen D, Kim T, Cui Y, et al. The novel long noncoding RNA u50535 promotes colorectal cancer growth and metastasis by regulating CCL20. Cell Death Dis(2018) 9(7):751. doi: 10.1038/s41419-018-0771-y

77. Samir N, Matboli M, El-Tayeb H, El-Tawdi A, Hassan MK, Waly A, et al. Competing endogenous RNA network crosstalk reveals novel molecular markers in colorectal cancer. J Cell Biochem(2018) 119(8):6869-81. doi: 10.1002/jcb.26884

78. Wang X, Liu F, Liu X, Wang F, Liao X, Chen Y, et al. Long non-coding RNA expression profiles reveals AK098783 is a biomarker to predict poor prognosis in patients with colorectal cancer. Jpn J Clin Oncol(2018) 48(5):480-4. doi: 10.1093/jjco/hyy037

79. Li Y, Zhao L, Zhang Y, Guan L, Zhang H, Zhou H, et al. Downregulation of the long non-coding RNA XLOC_010588 inhibits the invasion and migration of colorectal cancer. Oncol Rep(2018) 39(4):1619-30. doi: 10.3892/or.2018.6260

80. Gao T, Liu X, He B, Nie Z, Zhu C, Zhang P, et al. Exosomal lncRNA 91H is associated with poor development in colorectal cancer by modifying HNRNPK expression. Cancer Cell Int(2018) 18:11. doi: 10.1186/s12935-018-0506-2

81. Zhu Y, Li B, Liu Z, Jiang L, Wang G, Lv M, et al. Up-regulation of lncRNA SNHG1 indicates poor prognosis and promotes cell proliferation and metastasis of colorectal cancer by activation of the Wnt/β-catenin signaling pathway. Oncotarget(2017) 8(67):111715-27. doi: 10.18632/oncotarget.22903

82. Sun Z-Q, Chen C, Zhou Q-B, Liu J-B, Yang S-X, Li Z, et al. Long non-coding RNA LINC00959 predicts colorectal cancer patient prognosis and inhibits tumor progression. Oncotarget(2017) 8(57):97052-60. doi: 10.18632/oncotarget.21171

83. Wu S, Liu J, Wang X, Li M, Chen Z, Tang Y. Aberrant Expression of the Long Non-coding RNA GHRLOS and Its Prognostic Significance in Patients with Colorectal Cancer. J Cancer(2017) 8(19):4040-7. doi: 10.7150/jca.21304

84. Zhang W, Yuan W, Song J, Wang S, Gu X. LncRna CPS1-IT1 Suppresses Cell Proliferation, Invasion and Metastasis in Colorectal Cancer. Cell Physiol Biochem(2017) 44(2):567-80. doi: 10.1159/000485091

85. Zhang X, Xiong Y, Tang F, Bian Y, Chen Y, Zhang F. Long noncoding RNA HNF1A-AS1 indicates a poor prognosis of colorectal cancer and promotes carcinogenesis via activation of the Wnt/beta-catenin signaling pathway. Biomed Pharmacother(2017) 96:877-83. doi: 10.1016/j.biopha.2017.10.033

86. Tong GL, Wu X, Cheng BR, Li L, Li X, Li Z, et al. Knockdown of HOXA-AS2 suppresses proliferation and induces apoptosis in colorectal cancer. Am J Transl Res(2017) 9(10):4545-52. doi:

87. Cui C, Zhai D, Cai L, Duan Q, Xie L, Yu J. Long Noncoding RNA HEIH Promotes Colorectal Cancer Tumorigenesis via Counteracting miR-939Mediated Transcriptional Repression of Bcl-xL. Cancer Res Treat(2018) 50(3):992-1008. doi: 10.4143/crt.2017.226

88. He XS, Guo LC, Du MZ, Huang S, Huang RP, Zhan SH, et al. The long non-coding RNA NONHSAT062994 inhibits colorectal cancer by inactivating Akt signaling. Oncotarget(2017) 8(40):68696-706. doi: 10.18632/oncotarget.19827

89. Shen X, Bai Y, Luo B, Zhou X. Upregulation of lncRNA BANCR associated with the lymph node metastasis and poor prognosis in colorectal cancer. Biol Res(2017) 50(1):32. doi: 10.1186/s40659-017-0136-5

90. Lian YF, Yan CS, Ding J, Xia R, Ma ZH, Hui BQ, et al. A novel lncRNA, LL22NC03-N64E9.1, represses KLF2 transcription through binding with EZH2 in colorectal cancer. Oncotarget(2017) 8(35):59435-45. doi: 10.18632/oncotarget.19738

91. Xie F, Xiang XD, Huang QL, Ran PZ, Yuan YC, Li Q, et al. Reciprocal control of lncRNA-BCAT1 and β-catenin pathway reveals lncRNA-BCAT1 long non-coding RNA acts as a tumor suppressor in colorectal cancer. Oncotarget(2017) 8(14):23628-37. doi: 10.18632/oncotarget.15466

92. Dai M, Chen X, Mo S, Li J, Huang Z, Huang S, et al. Meta-signature LncRNAs serve as novel biomarkers for colorectal cancer: integrated bioinformatics analysis, experimental validation and diagnostic evaluation. Sci Rep(2017) 7:46572. doi: 10.1038/srep46572

93. Gao X, Wen J, Gao P, Zhang G, Zhang G. Overexpression of the long non-coding RNA, linc-UBC1, is associated with poor prognosis and facilitates cell proliferation, migration, and invasion in colorectal cancer. Onco Targets Ther(2017) 10:1017-26. doi: 10.2147/OTT.S129343

94. Wang JZ, Xu CL, Wu H, Shen SJ. LncRNA SNHG12 promotes cell growth and inhibits cell apoptosis in colorectal cancer cells. Braz J Med Biol Res(2017) 50(3):e6079. doi: 10.1590/1414-431X20176079

95. Tan W, Song ZZ, Xu Q, Qu X, Li Z, Wang Y, et al. Up-Regulated Expression of SPRY4-IT1 Predicts Poor Prognosis in Colorectal Cancer. Med Sci Monit(2017) 23:309-14. doi: 10.12659/msm.898369

96. Li T, Xu C, Cai B, Zhang M, Gao F, Gan J. Expression and clinicopathological significance of the lncRNA HOXA11-AS in colorectal cancer. Oncol Lett(2016) 12(5):4155-60. doi: 10.3892/ol.2016.5129

97. Liu T, Zhang X, Gao S, Jing F, Yang Y, Du L, et al. Exosomal long noncoding RNA CRNDE-h as a novel serum-based biomarker for diagnosis and prognosis of colorectal cancer. Oncotarget(2016) 7(51):85551-63. doi: 10.18632/oncotarget.13465

98. Yang L, Wei H, Xiao HJ. Long non-coding RNA Loc554202 expression as a prognostic factor in patients with colorectal cancer. Eur Rev Med Pharmacol Sci(2016) 20(20):4243-7. doi:

99. Li J, Lian Y, Yan C, Cai Z, Ding J, Ma Z, et al. Long non-coding RNA FOXP4-AS1 is an unfavourable prognostic factor and regulates proliferation and apoptosis in colorectal cancer. Cell Prolif(2017) 50(1)doi: 10.1111/cpr.12312

100. Wan L, Kong J, Tang J, Wu Y, Xu E, Lai M, et al. HOTAIRM1 as a potential biomarker for diagnosis of colorectal cancer functions the role in the tumour suppressor. J Cell Mol Med(2016) 20(11):2036-44. doi: 10.1111/jcmm.12892

101. Chen X, Zhu H, Wu X, Xie X, Huang G, Xu X, et al. Downregulated pseudogene CTNNAP1 promote tumor growth in human cancer by downregulating its cognate gene CTNNA1 expression. Oncotarget(2016) 7(34):55518-28. doi: 10.18632/oncotarget.10833

102. Kong J, Sun W, Li C, Wan L, Wang S, Wu Y, et al. Long non-coding RNA LINC01133 inhibits epithelial-mesenchymal transition and metastasis in colorectal cancer by interacting with SRSF6. Cancer Lett(2016) 380(2):476-84. doi: 10.1016/j.canlet.2016.07.015

103. Zhang Z, Zhou C, Chang Y, Zhang Z, Hu Y, Zhang F, et al. Long non-coding RNA CASC11 interacts with hnRNP-K and activates the WNT/beta-catenin pathway to promote growth and metastasis in colorectal cancer. Cancer Lett(2016) 376(1):62-73. doi: 10.1016/j.canlet.2016.03.022

104. Sun J, Ding C, Yang Z, Liu T, Zhang X, Zhao C, et al. The long non-coding RNA TUG1 indicates a poor prognosis for colorectal cancer and promotes metastasis by affecting epithelial-mesenchymal transition. J Transl Med(2016) 14:42. doi: 10.1186/s12967-016-0786-z

105. Yang L, Qiu M, Xu Y, Wang J, Zheng Y, Li M, et al. Upregulation of long non-coding RNA PRNCR1 in colorectal cancer promotes cell proliferation and cell cycle progression. Oncol Rep(2016) 35(1):318-24. doi: 10.3892/or.2015.4364
