# Supplementary material for: Biomarkers (mRNAs and Non-Coding RNAs) for the Diagnosis and Prognosis of Colorectal Cancer – From the Body Fluid to Tissue Level
Source: Front Oncol. 2021 Apr 29;11:632834. doi: 10.3389/fonc.2021.632834 (PMC8118670; doi:10.3389/fonc.2021.632834)
Supplement: Supplementary file 3 [file DataSheet_3.docx]

**Supplementary material 3**

**References(As showed in table 3.circRNAs as biomarkers for the diagnosis and prognosis of CRC)**

1. Chen MS, Lin CH, Huang LY, Qiu XM. CircRNA SMARCC1 Sponges MiR-140-3p to Regulate Cell Progression in Colorectal Cancer. *Cancer Manag Res*(2020*)* 12*:*4899-910. doi: 10.2147/CMAR.S254185

2. Xie Y, Li J, Li P, Li N, Zhang Y, Binang H, et al. RNA-Seq Profiling of Serum Exosomal Circular RNAs Reveals Circ-PNN as a Potential Biomarker for Human Colorectal Cancer. *Front Oncol*(2020*)* 10*:*982. doi: 10.3389/fonc.2020.00982

3. Li YF, Pei FL, Cao MZ. CircRNA_101951 promotes migration and invasion of colorectal cancer cells by regulating the KIF3A-mediated EMT pathway. *Exp Ther Med*(2020*)* 19*(*5)*:*3355-61. doi: 10.3892/etm.2020.8600

4. Yang H, Li X, Meng Q, Sun H, Wu S, Hu W, et al. CircPTK2 (hsa_circ_0005273) as a novel therapeutic target for metastatic colorectal cancer. *Mol Cancer*(2020*)* 19*(*1)*:*13. doi: 10.1186/s12943-020-1139-3

5. Zhou C, Liu H, Wang F, Hu T, Liang Z, Lan N, et al. circCAMSAP1 Promotes Tumor Growth in Colorectal Cancer via the miR-328-5p/E2F1 Axis. *Mol Ther*(2020*)* 28*(*3)*:*914-28. doi: 10.1016/j.ymthe.2019.12.008

6. Pan B, Qin J, Liu X, He B, Wang X, Pan Y, et al. Identification of Serum Exosomal hsa-circ-0004771 as a Novel Diagnostic Biomarker of Colorectal Cancer. *Front Genet*(2019*)* 10*:*1096. doi: 10.3389/fgene.2019.01096

7. Hon KW, Ab-Mutalib NS, Abdullah NMA, Jamal R, Abu N. Extracellular Vesicle-derived circular RNAs confers chemoresistance in Colorectal cancer. *Sci Rep*(2019*)* 9*(*1)*:*16497. doi: 10.1038/s41598-019-53063-y

8. Ye DX, Wang SS, Huang Y, Chi P. A 3-circular RNA signature as a noninvasive biomarker for diagnosis of colorectal cancer. *Cancer Cell Int*(2019*)* 19*:*276. doi: 10.1186/s12935-019-0995-7

9. Lin J, Cai D, Li W, Yu T, Mao H, Jiang S, et al. Plasma circular RNA panel acts as a novel diagnostic biomarker for colorectal cancer. *Clin Biochem*(2019*)* 74*:*60-8. doi: 10.1016/j.clinbiochem.2019.10.012

10. Yang G, Zhang T, Ye J, Yang J, Chen C, Cai S, et al. Circ-ITGA7 sponges miR-3187-3p to upregulate ASXL1, suppressing colorectal cancer proliferation. *Cancer Manag Res*(2019*)* 11*:*6499-509. doi: 10.2147/CMAR.S203137

11. Tian J, Xi X, Wang J, Yu J, Huang Q, Ma R, et al. CircRNA hsa_circ_0004585 as a potential biomarker for colorectal cancer. *Cancer Manag Res*(2019*)* 11*:*5413-23. doi: 10.2147/CMAR.S199436

12. Ge J, Jin Y, Lv X, Liao Q, Luo C, Ye G, et al. Expression profiles of circular RNAs in human colorectal cancer based on RNA deep sequencing. *J Clin Lab Anal*(2019*)* 33*(*7)*:*e22952. doi: 10.1002/jcla.22952

13. Li XN, Wang ZJ, Ye CX, Zhao BC, Huang XX, Yang L. Circular RNA circVAPA is up-regulated and exerts oncogenic properties by sponging miR-101 in colorectal cancer. *Biomed Pharmacother*(2019*)* 112*:*108611. doi: 10.1016/j.biopha.2019.108611

14. Li XN, Wang ZJ, Ye CX, Zhao BC, Li ZL, Yang Y. RNA sequencing reveals the expression profiles of circRNA and indicates that circDDX17 acts as a tumor suppressor in colorectal cancer. *J Exp Clin Cancer Res*(2018*)* 37*(*1)*:*325. doi: 10.1186/s13046-018-1006-x

15. Yuan Y, Liu W, Zhang Y, Zhang Y, Sun S. CircRNA circ_0026344 as a prognostic biomarker suppresses colorectal cancer progression via microRNA-21 and microRNA-31. *Biochem Biophys Res Commun*(2018*)* 503*(*2)*:*870-5. doi: 10.1016/j.bbrc.2018.06.089

16. Zeng K, Chen X, Xu M, Liu X, Hu X, Xu T, et al. CircHIPK3 promotes colorectal cancer growth and metastasis by sponging miR-7. *Cell Death Dis*(2018*)* 9*(*4)*:*417. doi: 10.1038/s41419-018-0454-8

17. Zhang W, Yang S, Liu Y, Wang Y, Lin T, Li Y, et al. Hsa_circ_0007534 as a blood-based marker for the diagnosis of colorectal cancer and its prognostic value. *Int J Clin Exp Pathol*(2018*)* 11*(*3)*:*1399-406. doi:

18. Zhuo F, Lin H, Chen Z, Huang Z, Hu J. The expression profile and clinical significance of circRNA0003906 in colorectal cancer. *Onco Targets Ther*(2017*)* 10*:*5187-93. doi: 10.2147/OTT.S147378

19. Zhang P, Zuo Z, Shang W, Wu A, Bi R, Wu J, et al. Identification of differentially expressed circular RNAs in human colorectal cancer. *Tumour Biol*(2017*)* 39*(*3)*:*1010428317694546. doi: 10.1177/1010428317694546

20. Xie H, Ren X, Xin S, Lan X, Lu G, Lin Y, et al. Emerging roles of circRNA_001569 targeting miR-145 in the proliferation and invasion of colorectal cancer. *Oncotarget*(2016*)* 7*(*18)*:*26680-91. doi: 10.18632/oncotarget.8589

21. Zhong D, Li P, Gong PY. Hsa_circ_0005075 promotes the proliferation and invasion of colorectal cancer cells. *Int J Biol Markers*(2019*)* 34*(*3)*:*284-91. doi: 10.1177/1724600819872765

22. Zhang XL, Xu LL, Wang F. Hsa_circ_0020397 regulates colorectal cancer cell viability, apoptosis and invasion by promoting the expression of the miR-138 targets TERT and PD-L1. *Cell Biol Int*(2017*)* 41*(*9)*:*1056-64. doi: 10.1002/cbin.10826

23. Jin C, Wang A, Liu L, Wang G, Li G. Hsa_circ_0136666 promotes the proliferation and invasion of colorectal cancer through miR-136/SH2B1 axis. *J Cell Physiol*(2019*)* 234*(*5)*:*7247-56. doi: 10.1002/jcp.27482

24. Jin Y, Yu LL, Zhang B, Liu CF, Chen Y. Circular RNA hsa_circ_0000523 regulates the proliferation and apoptosis of colorectal cancer cells as miRNA sponge. *Braz J Med Biol Res*(2018*)* 51*(*12)*:*e7811. doi: 10.1590/1414-431X20187811

25. Zhang J, Liu H, Zhao P, Zhou H, Mao T. Has_circ_0055625 from circRNA profile increases colon cancer cell growth by sponging miR-106b-5p. *J Cell Biochem*(2019*)* 120*(*3)*:*3027-37. doi: 10.1002/jcb.27355

26. Min L, Wang H, Zeng Y. CircRNA_104916 regulates migration, apoptosis and epithelial-mesenchymal transition in colon cancer cells. *Front Biosci (Landmark Ed)*(2019*)* 24*:*819-32. doi:

27. Zheng X, Chen L, Zhou Y, Wang Q, Zheng Z, Xu B, et al. A novel protein encoded by a circular RNA circPPP1R12A promotes tumor pathogenesis and metastasis of colon cancer via Hippo-YAP signaling. *Mol Cancer*(2019*)* 18*(*1)*:*47. doi: 10.1186/s12943-019-1010-6

28. Ji W, Qiu C, Wang M, Mao N, Wu S, Dai Y. Hsa_circ_0001649: A circular RNA and potential novel biomarker for colorectal cancer. *Biochem Biophys Res Commun*(2018*)* 497*(*1)*:*122-6. doi: 10.1016/j.bbrc.2018.02.036

29. Wang J, Li X, Lu L, He L, Hu H, Xu Z. Circular RNA hsa_circ_0000567 can be used as a promising diagnostic biomarker for human colorectal cancer. *J Clin Lab Anal*(2018*)* 32*(*5)*:*e22379. doi: 10.1002/jcla.22379

30. Zhang Z, Song N, Wang Y, Zhong J, Gu T, Yang L, et al. Analysis of differentially expressed circular RNAs for the identification of a coexpression RNA network and signature in colorectal cancer. *Journal of Cellular Biochemistry*(2018*)* 120*(*4)*:*6409-19. doi: 10.1002/jcb.27928

31. Lu H, Yao B, Wen X, Jia B. FBXW7 circular RNA regulates proliferation, migration and invasion of colorectal carcinoma through NEK2, mTOR, and PTEN signaling pathways in vitro and in vivo. *BMC Cancer*(2019*)* 19*(*1)*:*918. doi: 10.1186/s12885-019-6028-z

32. Ge Z, Li LF, Wang CY, Wang Y, Ma WL. CircMTO1 inhibits cell proliferation and invasion by regulating Wnt/β-catenin signaling pathway in colorectal cancer. *Eur Rev Med Pharmacol Sci*(2018*)* 22*(*23)*:*8203-9. doi: 10.26355/eurrev_201812_16513

33. Ruan H, Deng X, Dong L, Yang D, Xu Y, Peng H, et al. Circular RNA circ_0002138 is down-regulated and suppresses cell proliferation in colorectal cancer. *Biomed Pharmacother*(2019*)* 111*:*1022-8. doi: 10.1016/j.biopha.2018.12.150

34. Yang N, Xu B, Kong P, Han M, Li BH. Hsa_circ_0002320: a novel clinical biomarker for colorectal cancer prognosis. *Medicine (Baltimore)*(2020*)* 99*(*28)*:*e21224. doi: 10.1097/MD.0000000000021224

35. Li J, Ni S, Zhou C, Ye M. The expression profile and clinical application potential of hsa_circ_0000711 in colorectal cancer. *Cancer Manag Res*(2018*)* 10*:*2777-84. doi: 10.2147/CMAR.S172388

36. He C, Huang C, Zhou R, Yu H. CircLMNB1 promotes colorectal cancer by regulating cell proliferation, apoptosis and epithelial-mesenchymal transition. *OncoTargets and Therapy*(2019*)* Volume 12*:*6349-59. doi: 10.2147/ott.S204741

37. Xiao YS, Tong HZ, Yuan XH, Xiong CH, Xu XY, Zeng YF. CircFADS2: A potential prognostic biomarker of colorectal cancer. *Exp Biol Med (Maywood)*(2020*)* 245*(*14)*:*1233-41. doi: 10.1177/1535370220929965

38. Dai J, Zhuang Y, Tang M, Qian Q, Chen JP. CircRNA UBAP2 facilitates the progression of colorectal cancer by regulating miR-199a/VEGFA pathway. *Eur Rev Med Pharmacol Sci*(2020*)* 24*(*15)*:*7963-71. doi: 10.26355/eurrev_202008_22479

39. Jing L, Wu J, Tang X, Ma M, Long F, Tian B, et al. Identification of circular RNA hsa_circ_0044556 and its effect on the progression of colorectal cancer. *Cancer Cell Int*(2020*)* 20*:*427. doi: 10.1186/s12935-020-01523-1

40. Zhang X, Xu Y, Yamaguchi K, Hu J, Zhang L, Wang J, et al. Circular RNA circVAPA knockdown suppresses colorectal cancer cell growth process by regulating miR-125a/CREB5 axis. *Cancer Cell Int*(2020*)* 20*:*103. doi: 10.1186/s12935-020-01178-y

41. Chen H, Li X, Ye C, Chen Z, Wang Z. Circular RNA circHUWE1 Is Upregulated and Promotes Cell Proliferation, Migration and Invasion in Colorectal Cancer by Sponging miR-486. *Onco Targets Ther*(2020*)* 13*:*423-34. doi: 10.2147/OTT.S233338

42. Tang X, Sun G, He Q, Wang C, Shi J, Gao L, et al. Circular noncoding RNA circMBOAT2 is a novel tumor marker and regulates proliferation/migration by sponging miR-519d-3p in colorectal cancer. *Cell Death Dis*(2020*)* 11*(*8)*:*625. doi:
