# Supplementary material for: Biomarkers (mRNAs and Non-Coding RNAs) for the Diagnosis and Prognosis of Colorectal Cancer – From the Body Fluid to Tissue Level
Source: Front Oncol. 2021 Apr 29;11:632834. doi: 10.3389/fonc.2021.632834 (PMC8118670; doi:10.3389/fonc.2021.632834)
Supplement: Supplementary file 4 [file DataSheet_4.docx]

**Supplementary material 4**

**References(As showed in table 4. piRNAs as potential biomarker for colorectal cancer)**

1. Okugawa Y, Toiyama Y, Toden S, Mitoma H, Nagasaka T, Tanaka K, et al. Clinical significance of SNORA42 as an oncogene and a prognostic biomarker in colorectal cancer. *Gut*(2017*)* 66*(*1)*:*107-17. doi: 10.1136/gutjnl-2015-309359

2. Yoshida K, Toden S, Weng W, Shigeyasu K, Miyoshi J, Turner J, et al. SNORA21 - An Oncogenic Small Nucleolar RNA, with a Prognostic Biomarker Potential in Human Colorectal Cancer. *EBioMedicine*(2017*)* 22*:*68-77. doi: 10.1016/j.ebiom.2017.07.009

3. Fang X, Yang D, Luo H, Wu S, Dong W, Xiao J, et al. SNORD126 promotes HCC and CRC cell growth by activating the PI3K-AKT pathway through FGFR2. *J Mol Cell Biol*(2017*)* 9*(*3)*:*243-55. doi: 10.1093/jmcb/mjw048

4. Wu L, Zheng J, Chen P, Liu Q, Yuan Y. Small nucleolar RNA ACA11 promotes proliferation, migration and invasion in hepatocellular carcinoma by targeting the PI3K/AKT signaling pathway. *Biomed Pharmacother*(2017*)* 90*:*705-12. doi: 10.1016/j.biopha.2017.04.014

5. Tang G, Zeng Z, Sun W, Li S, You C, Tang F, et al. Small Nucleolar RNA 71A Promotes Lung Cancer Cell Proliferation, Migration and Invasion via MAPK/ERK Pathway. *J Cancer*(2019*)* 10*(*10)*:*2261-75. doi: 10.7150/jca.31077

6. Zhang C, Zhao LM, Wu H, Tian G, Dai SL, Zhao RY, et al. C/D-Box Snord105b Promotes Tumorigenesis in Gastric Cancer via ALDOA/C-Myc Pathway. *Cell Physiol Biochem*(2018*)* 45*(*6)*:*2471-82. doi: 10.1159/000488265

7. Sun Y, Chen E, Li Y, Ye D, Cai Y, Wang Q, et al. H/ACA box small nucleolar RNA 7B acts as an oncogene and a potential prognostic biomarker in breast cancer. *Cancer Cell Int*(2019*)* 19*:*125. doi: 10.1186/s12935-019-0830-1

8. Yi C, Wan X, Zhang Y, Fu F, Zhao C, Qin R, et al. SNORA42 enhances prostate cancer cell viability, migration and EMT and is correlated with prostate cancer poor prognosis. *Int J Biochem Cell Biol*(2018*)* 102*:*138-50. doi: 10.1016/j.biocel.2018.07.009
