# Supplementary material for: Biomarkers (mRNAs and Non-Coding RNAs) for the Diagnosis and Prognosis of Colorectal Cancer – From the Body Fluid to Tissue Level
Source: Front Oncol. 2021 Apr 29;11:632834. doi: 10.3389/fonc.2021.632834 (PMC8118670; doi:10.3389/fonc.2021.632834)
Supplement: Supplementary file 5 [file DataSheet_5.docx]

**Supplementary material 5**

**References (As showed in table 5. piRNAs as potential biomarker for colorectal cancer)**

1. Weng W, Liu N, Toiyama Y, Kusunoki M, Nagasaka T, Fujiwara T, et al. Novel evidence for a PIWI-interacting RNA (piRNA) as an oncogenic mediator of disease progression, and a potential prognostic biomarker in colorectal cancer. *Mol Cancer*(2018*)* 17*(*1)*:*16. doi: 10.1186/s12943-018-0767-3

2. Qu A, Wang W, Yang Y, Zhang X, Dong Y, Zheng G, et al. A serum piRNA signature as promising non-invasive diagnostic and prognostic biomarkers for colorectal cancer. *Cancer Manag Res*(2019*)* 11*:*3703-20. doi: 10.2147/CMAR.S193266

3. Feng J, Yang M, Wei Q, Song F, Zhang Y, Wang X, et al. Novel evidence for oncogenic piRNA-823 as a promising prognostic biomarker and a potential therapeutic target in colorectal cancer. *J Cell Mol Med*(2020*)* doi: 10.1111/jcmm.15537

4. Wang Z, Yang H, Ma D, Mu Y, Tan X, Hao Q, et al. Serum PIWI-Interacting RNAs piR-020619 and piR-020450 Are Promising Novel Biomarkers for Early Detection of Colorectal Cancer. *Cancer Epidemiol Biomarkers Prev*(2020*)* 29*(*5)*:*990-8. doi: 10.1158/1055-9965.EPI-19-1148

5. Iyer DN, Wan TM, Man JH, Sin RW, Li X, Lo OS, et al. Small RNA Profiling of piRNAs in Colorectal Cancer Identifies Consistent Overexpression of piR-24000 That Correlates Clinically with an Aggressive Disease Phenotype. *Cancers (Basel)*(2020*)* 12*(*1)doi: 10.3390/cancers12010188

6. Mai D, Ding P, Tan L, Zhang J, Pan Z, Bai R, et al. PIWI-interacting RNA-54265 is oncogenic and a potential therapeutic target in colorectal adenocarcinoma. *Theranostics*(2018*)* 8*(*19)*:*5213-30. doi: 10.7150/thno.28001
